# Probing 3‐Amino‐2*H*‐Azaindazoles as Allosteric Inhibitors of the Protein Tyrosine Phosphatase SHP2

**DOI:** 10.1002/cmdc.70341

**Published:** 2026-06-14

**Authors:** Machoud Amoussa, Nina‐Louisa Efrém, Feng Li, Ziqiong Guo, Yvette Roske, Katrin Jana Frank, Szymon Pach, Clemens Alexander Wolf, Feng Bo, Marina Lesina, Mika Kintzel, Rana Alsalim, Victoria Zeitz, Noémi Csorba, Silke Radetzki, György M. Keserű, Oliver Daumke, Hana Algül, Gerhard Wolber, Jia Li, Marc Nazaré

**Affiliations:** ^1^ Leibniz‐Forschungsinstitut für Molekulare Pharmakologie (FMP) Campus Berlin‐Buch Berlin Germany; ^2^ Freie Universität Molecular Design Group Institute of Pharmacy Berlin Germany; ^3^ Karlsruhe Institute of Technology Organic Chemistry I Institute of Organic Chemistry Karlsruhe Germany; ^4^ School of Chinese Materia Medica Nanjing University of Chinese Medicine Nanjing China; ^5^ State Key Laboratory of Chemical Biology Shanghai Institute of Materia Medica (SIMM) Chinese Academy of Sciences Pudong Shanghai China; ^6^ Max Delbrück Center for Molecular Medicine (MDC) Structural Biology Campus Berlin‐Buch Berlin Germany; ^7^ Comprehensive Cancer Center München Institute for Tumor Metabolism Klinikum rechts der Isar Technical University of Munich School of Medicine and Health Munich Bavaria Germany; ^8^ Department of Pharmaceutical Chemistry Philipps‐Universität Marburg Marburg Germany; ^9^ Research Centre for Natural Sciences (RCNS) Budapest Hungary; ^10^ National Laboratory for Drug Research and Development Budapest Hungary; ^11^ Department of Organic Chemistry and Technology Faculty of Chemical Technology and Biotechnology Budapest University of Technology and Economics Budapest Hungary; ^12^ Department of Chemistry Organic and Bioorganic Chemistry Bielefeld University Bielefeld Germany

**Keywords:** 2*H*‐indazoles, allosteric inhibition, heterocycles, phosphatases, SHP2

## Abstract

Src homology 2‐containing protein tyrosine phosphatase 2 (SHP2) is an attractive therapeutic target in oncology and immunology‐related disorders. However, developing novel phosphatase inhibitors that combine high potency, selectivity, cellular permeability, and drug‐like properties remains challenging. The discovery of an allosteric mode of inhibition for SHP2 was a breakthrough, enabling the development of selective inhibitors that stabilize the phosphatase in its inactive conformation. We identified 2*H*‐indazoles as a privileged and underexplored scaffold. Using our recently described palladium‐catalyzed domino reaction as a key synthetic step, 3‐amino‐2*H*‐indazoles were efficiently accessed from readily available precursors, enabling rapid exploration of novel allosteric inhibitors of SHP2. This approach led to compound **17g**, a potent and selective allosteric SHP2 inhibitor (SHP2^WT^ IC_50_ = 49 nM). High‐resolution structural characterization by X‐ray crystallography revealed binding within the SHP2 allosteric tunnel. Consistent with its biological activity, compound **17g** also effectively suppressed ERK phosphorylation in MV‐4‐11, Panc‐1, and KYSE520 cells with an IC_50_ of 50, 250, and 410 nM, respectively. These findings not only highlight the therapeutic potential of 2*H*‐azaindazoles as a new class of SHP2 inhibitors but also underscore the importance of advances in efficient synthetic methodologies for constructing novel heterocyclic scaffolds and substitution patterns.

## Graphical abstract

A palladium‐catalyzed domino reaction facilitated the discovery and rapid synthesis of selective, potent 3‐amino‐2H‐azaindazole derivatives serving as allosteric inhibitors of SHP2. Structural analysis via co‐crystallography confirmed binding at the allosteric site, thereby stabilizing the autoinhibited SHP2 conformation, qualifying this scaffold as a promising basis for the development of allosteric SHP2 inhibitors.

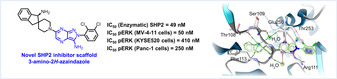

## 1 Introduction

**FIGURE 1:**
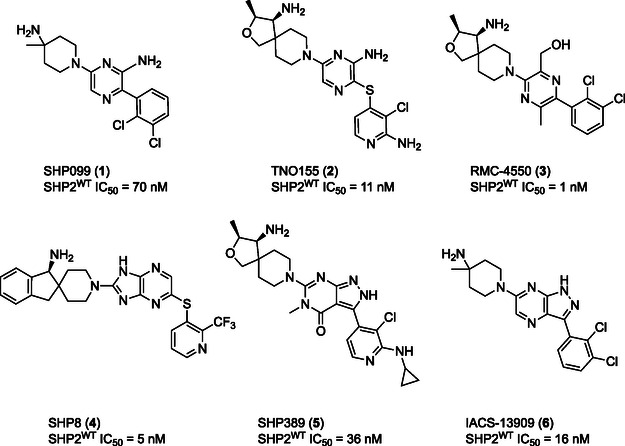
Selection of described allosteric SHP2 inhibitors. SHP099 **1** [9], TNO155 **2** [10], RMC‐4550 **3** [12], SHP8 **4** [14], SHP389 **5** [15], IACS‐13909 **6** [13]**.** Reported IC_50_ values were taken from the cited literature.

SHP2 inhibition has emerged as a promising pharmacological approach for the development of antitumor therapies [1, 2, 3]. As a nonreceptor protein tyrosine phosphatase, SHP2 is encoded by the proto‐oncogene protein tyrosine phosphatase nonreceptor type 11 (*PTPN11*) and plays an essential role in multiple signaling pathways, including the Ras/ERK, PI3K/AKT, and JAK/STAT pathways [4, 5, 6]. Furthermore, SHP2 is involved in the immunomodulatory programmed cell death protein 1 (PD‐1)/programmed death‐ligand 1 (PD‐L1) pathway, and other signaling pathways upon stimulation by growth factors [7, 8]. Since the discovery of the first allosteric SHP2 inhibitor SHP099 **1** [9], there have been extensive investigations and significant advancements in designing allosteric inhibitors that target a specific tunnel‐like allosteric site. These efforts provided several novel chemotypes, such as TNO155 **2** [10], RMC‐4550 **3** [11, 12], IACS‐13909 **6** [13], some of which are currently undergoing clinical trials for the treatment of solid tumors (Figure 1). While these allosteric SHP2 inhibitors act as molecular glues that stabilize SHP2 in its inactive autoinhibited conformation, they still encounter challenges, such as resistance mutations and suboptimal pharmacokinetics [16]. This highlights the need to explore new scaffolds with improved properties.

Given the wealth of available data, including crystal structures, and the identified privileged pharmacophore groups associated with allosteric SHP2 inhibitors, we aimed to explore the potential to develop novel allosteric SHP2 inhibitors. We hypothesized that the 3‐amino‐2*H*‐azaindazole heterocyclic core could serve as a novel scaffold for allosteric inhibition of SHP2.

**FIGURE 2:**
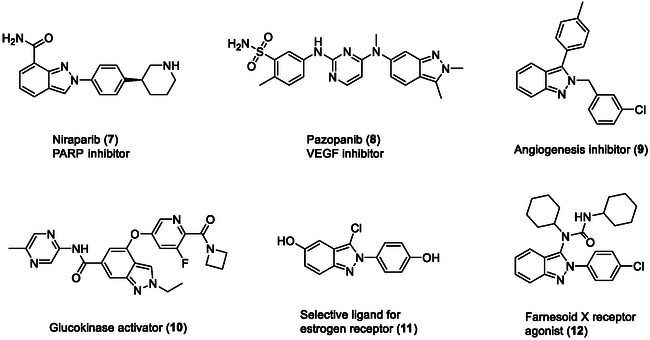
Pharmacologically active molecules bearing the 2*H*‐indazole scaffold. Niraparib **7** [19, 20], Pazopanib **8** [21, 22], angiogenesis inhibitor **9** [23], glucokinase activator **10** [24], selective ligand for estrogen receptor 11 [25], and farnesoid X receptor agonist 12 [26].

Indeed, the facile regioselective construction to access diverse heterocyclic scaffolds is at the center of many medicinal chemistry lead finding efforts, often hampered by the paucity of appropriate methodologies requiring multistep synthesis. Among these heterocycles, 2*H*‐indazoles have been successively gaining importance as a privileged scaffold [17, 18]. 2*H*‐indazoles were identified as key substructures that play a vital role in mediating biological activity against various biological targets as well as featuring good PK properties, culminating in successful drug candidates such as Niraparib **7** [19, 20], a selective PARP‐1/2 inhibitor, and Pazopanib **8** [21, 22], a multi‐targeted tyrosine kinase inhibitor (Figure 2).

Due to the significance of the 2*H*‐indazole scaffold, extensive efforts have been devoted to facilitating the synthesis of such motifs and substitution patterns [27, 28, 29, 30, 31, 32, 33]. Direct regioselective formation of 2*H*‐indazole derivatives appears to be challenging, and alternative approaches such as *N*‐alkylation or *N*‐arylation often lead to varying, difficult‐to‐separate mixtures of 1*H*‐ and 2*H*‐indazole regioisomers, in which the 1*H*‐indazole is the thermodynamically favored product [34]. Therefore, the reliable direct regioselective access to *N*‐substituted 2*H*‐indazoles remains an important goal both in medicinal chemistry and organic synthesis.

Among the most established approaches for the regioselective construction of 2*H*‐indazoles is the Cadogan cyclization, which involves the reductive cyclization of an aryl imine intermediate using phosphorus reagents [35, 36, 37, 38, 39, 40, 41]. Other examples include reductive cyclization of 2‐nitrobenzylamines with TiCl_4_/Zn [42], Fe‐catalyzed N─N bond formation of 2‐azidophenylketoximes [43], [3 + 2] cycloaddition of arynes and sydnones [44], Co(III)‐catalyzed C─H addition of azobenzenes to aldehydes [45], Rh(III)‐catalyzed C─H addition of azobenzenes with alpha‐keto aldehydes [46], and the Cu‐catalyzed reaction of 2‐bromobenzaldehydes with primary amines and sodium azides [47]. In recent years, several photocatalytic approaches have also been developed [48, 49, 50]. In addition, Pd‐catalyzed intramolecular domino reactions, based on amination reactions between hydrazines and 2‐halophenylacetylenes or 2‐halophenylnitriles, were developed to provide an efficient access to a wide variety of 2*H*‐indazoles and 3‐amino‐2*H*‐azaindazoles, respectively, as well as their aza congeners [23, 30, 51, 52].

Herein, we describe the design, synthesis, and structure–activity relationship (SAR) evaluation of various 3‐amino‐2*H*‐azaindazole‐based SHP2 inhibitors employing a scaffold hopping strategy. By utilizing a regioselective Pd‐catalyzed domino reaction recently developed by us, [23] we were able to efficiently access the 3‐amino‐2*H*‐indazole scaffold through the rapid, convergent two‐step synthesis of various analogs. We subsequently conducted SAR analysis on this new class of inhibitors, enabling the identification of a potent lead compound.

## 2 Results and Discussion

### 2.1 Scaffold Hopping Design Approach

Our re‐scaffolding strategy was based on two conceptual considerations. First, the application of the regioselective Pd‐catalyzed domino reaction for the rapid and convergent access of 3‐amino‐2*H*‐azaindazole‐based inhibitors, and secondly, the strong preservation of a common construction principle for most SHP2 inhibitors with two highly privileged distal substituents. To guide our design, we therefore analyzed available inhibitors reported to bind in the tunnel allosteric site of SHP2 [53, 54, 55].

**FIGURE 3:**
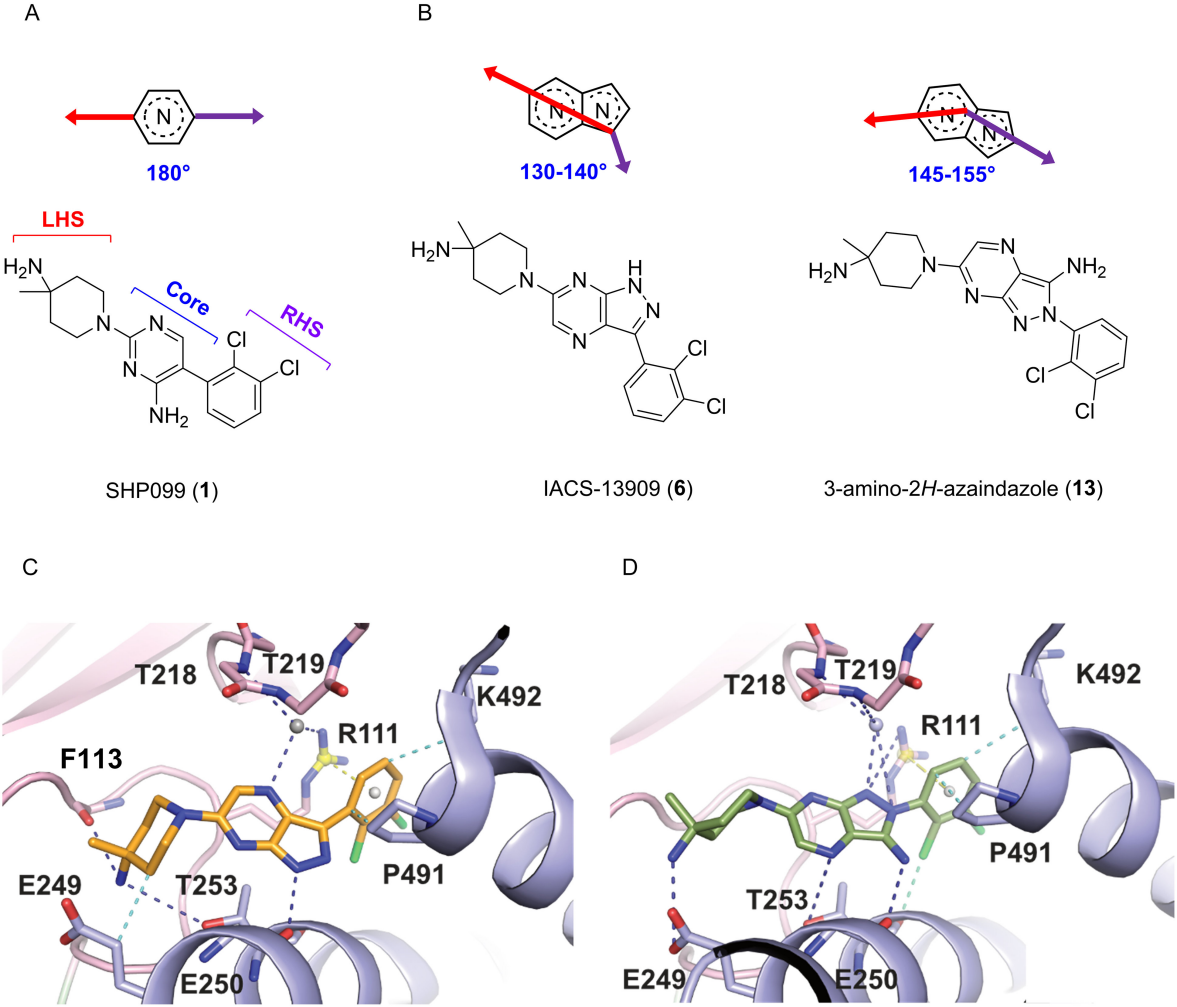
(A) Chemical structure of SHP099 **1** with the LHS, the central core and RHS; (B) Commonly observed geometry in allosteric SHP2 inhibitors IACS‐13909 **6** and the novel 3‐amino‐2*H*‐azaindazole **13**: in red and purple are the vectors directed to the LHS and RHS respectively with corresponding angle formed at their intersection; (C) Binding mode of IACS‐13909 **6** within SHP2 (PDB ID: 6WU8); (D) Docking pose of compound **13** in the SHP2 allosteric binding pocket (PDB ID: 6WU8). The hydrogen bonding interactions are denoted by dark blue dashed lines, π‐cation interactions by yellow lines, hydrophobic interactions by light blue lines, and halogen interactions by green lines.

Taking SHP099 **1** as a representative inhibitor, the compound can be structurally dissected into three parts: a halogenated aryl ring on the right‐hand side (RHS), a central amino‐substituted heteroaromatic ring, and an amino‐substituted heterocycle on the left‐hand side (LHS) (Figure 3A). This overall construction principle is found among most described allosteric inhibitors. While the LHS and RHS pharmacophores, due to their interaction with key residues in the allosteric site, are highly conserved substituents, further analysis revealed considerable diversity in the central scaffold. Examples include monocyclic cores such as furanyl amide derivatives [15] as well as fused bicyclic systems like imidazopyrazine [14] and 5‐azaquinaxoline [56]; supporting the rationale of our scaffold hopping strategy.

Interestingly, we observed that most reported inhibitors share a conserved vectorial orientation of the distal substituents. For instance, monocyclic core inhibitors such as SHP099 **1** and RMC‐4550 **3** exhibit a linear extension with an angle of 180° between the vectors extending from the central core to the LHS and RHS substituents, while fused bicyclic scaffolds such as IACS‐13909 **6** show a more angular geometry of 130°–140° (Figure 3B), suggesting underexplored geometry flexibility within the allosteric binding pocket**.** Guided by a scaffold replacement strategy, we hypothesized that the 3‐amino‐2*H*‐azaindazole framework could serve as a structurally distinct yet functionally analogous core, retaining key hydrogen bond interactions while potentially accessing alternative regions of the binding site. Based on these hypotheses, we designed compound **13**, bearing a 3‐amino‐2*H*‐azaindazole core that could adopt an angular geometry of 145°–155°, providing a framework for further probing the conformational flexibility of the SHP2 allosteric pocket.

To support our scaffold replacement strategy, we conducted docking studies using the Glide module (Schrödinger) based on the co‐crystal structure of IACS‐13909 **6** bound to SHP2 (PDB ID: 6WU8). The interactions between IACS‐13909 **6**, as well as for the docked compound **13**, in the allosteric binding site of SHP2 are shown in Figure 3C,D. When performing a structure‐based alignment of the co‐crystal structure of IACS‐13909 **6** bound to SHP2, the alignment revealed a high degree of spatial overlap, particularly between the central heterocyclic core and the aromatic aryl ring of both ligands (Figure S1). A notable distinction was observed in the LHS region, where the primary amine of compound **13** adopts a shifted orientation relative to the amine group in IACS‐13909 **6**, potentially enabling additional interactions with residues not previously targeted in the allosteric pocket. Analyzing key contacts revealed that according to our docking study, the aniline group and the nitrogen at position 1 of the 3‐amino‐2*H*‐pyrazol ring in compound **13** formed key hydrogen–bond interactions with Glu250 and Arg111, as well as water‐mediated hydrogen–bond interactions with Thr218 and Thr219, respectively, recapitulating the interaction pattern observed for IACS‐13909 **6**. However, unlike IACS‐13909 **6**, compound **13** lacked key hydrogen bond interactions mediated by the aliphatic amine with residues Phe113 and Glu110. Instead, we observed an unprecedented hydrogen bond between the aliphatic amine and Glu249. In addition, the 2,3‐dichlorophenyl group engaged in a halogen bond between the *ortho*‐chlorine and Thr253, another interaction absent in the IACS‐13909 **6** co‐crystal structure.

While docking scores were comparable between the two compounds, these structural insights supported compound **13** as a promising starting point for further optimization. As such, we prioritized the synthesis and subsequent SAR exploration at the LHS, RHS, and core regions.

### 2.2 Chemical Synthesis of 3‐Amino‐2*H*‐Azaindazole‐based Compounds

**SCHEME 1:**
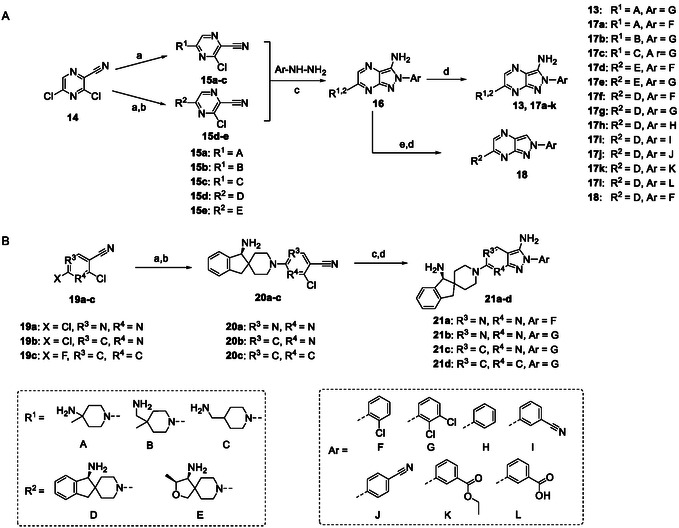
Chemical synthesis of targeted compounds. Reagents and conditions: (a) Boc‐protected/Boc‐unprotected amine, DCM, DIPEA, rt, 4 h, 45–85%; (b) Boc_2_O, DIPEA, DCM, rt, 6 h, 40–85% over two steps; (c) phenylhydrazine **F‐L**, Pd_2_(dba)_3_, TTBP · HBF_4_, Cs_2_CO_3_, 1,4‐dioxane, 112°C, 2–4 h; (d) TFA, DCM, rt, 1 h, 10–30% over two steps; (e) tBuONO, THF, 66°C, 34%.

The synthetic approach for preparing the 3‐amino‐2*H*‐azaindazole‐based series of compounds is depicted in Scheme 1. All 3‐amino‐2*H*‐azaindazole‐based SHP2 inhibitors were assembled by using a Pd‐catalyzed domino reaction described by Alsalim et al. [23] in a short convergent route.

Targeted compound **13** was synthesized starting from commercially available 3,5‐dichloropyrazine‐2‐carbonitrile **14**, which was subjected to a nucleophilic aromatic substitution reaction in the presence of *tert*‐butyl (4‐methylpiperidin‐4‐yl)carbamate to furnish the intermediate **15a**. **15a** was then directly coupled with the commercially available (2,3‐dichlorophenyl)hydrazine in the central palladium‐catalyzed domino reaction in the presence of Pd_2_(dba)_3_ to afford final compound **13** after Boc deprotection of the primary aliphatic amine with TFA in DCM.

Compounds **17a–k** were synthesized following the same strategy using the corresponding amines **A–E** and aryl hydrazine **F–L**, taking into account that intermediates **15d** and **15e** required an additional Boc protection step before proceeding with the Pd‐catalyzed reaction and subsequent TFA‐mediated Boc deprotection for accessing the final compounds **17a–k**.

Compound **18** was obtained after a reductive deamination of the aliphatic amine Boc‐protected intermediate in the presence of *t*BuONO in THF at 66°C, followed by a TFA‐mediated Boc deprotection.

Compounds **21a** to **21d** were obtained as described in Scheme 1B following the same procedure as compound **17b,** but starting with the commercially available 2,4‐dichloropyrimidine‐5‐carbonitrile **19a**, 2,6‐dichloronicotinonitrile **19b**, and 2‐chloro‐4‐fluorobenzonitrile **19c**, respectively.

Overall, this synthetic strategy enabled us to efficiently and successfully synthesize a diverse array of analogs of **13** through the application of a convergent building block approach. All three regions of the 3‐amino‐2*H*‐azaindazole‐based inhibitors were diversified: the LHS, the hydrazine building block to introduce novel RHS, and variation of the 2‐halobenzonitrile to modify the central scaffold.

### 2.3 Evaluation of SHP2 Inhibition

Next, we determined the inhibitory potency of the synthesized compounds against wild‐type (WT), full‐length SHP2^WT^. We used the well‐established fluorescence‐based phosphatase biochemical assay in the presence of an activating phosphotyrosine peptide and measured the dephosphorylation rate of 6,8‐difluoro‐4‐methylumbelliferyl phosphate (DiFMUP) [57, 58]. Our evaluation started with compound **13** and compound **17a,** respectively, carrying a 2,3‐dichlorophenyl and 2‐chlorophenyl moiety at the RHS. While compound **13** showed promising inhibition with an IC_50_ of 540 nM against SHP2, compound **17a** was surprisingly much less potent with an IC_50_ value above 10 µM against SHP2, illustrating the strong impact of the missing chlorine in the *meta* position of the phenyl ring Ar. Installing different privileged amines at the LHS, such as monocyclic, bicyclic, and tricyclic amino moieties while maintaining the 3‐amino‐2*H*‐azaindazole scaffold in combination with the 2‐chlorophenyl and 2,3‐dichlorophenyl moieties as RHS showed a very distinct activity trend (Table 1). Most of the modifications, such as replacing the primary amine in **13** by a methyl amine **17b** or removing the geminal methyl group **17c**, significantly reduced the SHP2 inhibitory activity from 540 nM to >10 µM. The spirocyclic ether (3*S*, 4*S*)−3,8‐dimethyl‐2‐oxa‐8‐azaspiro [4.5]decan‐4‐amine, furnished **17d** with an IC_50_ value >10 µM as most of the 2‐chlorophenyl derivatives, while **17e**, the matched pair bearing the 2,3‐dichlorophenyl moiety, yielded an IC_50_ value of 6,100 nM, showing more than an 11‐fold decrease in inhibitory activity compared to analog **13**. Notably, the addition of the (S)−1,3‐dihydrospiro[indene‐2,4′‐piperidin]‐1‐amine system enhanced the activity of compounds **17f** and **17g,** yielding IC_50_ values of 560 nM and 49 nM, respectively. As compounds **17a**, **17d,** and **17f** bearing the 2‐chlorophenyl moiety as RHS showed a considerable drop in potency compared to their respective matched pairs **13**, **17e,** and **17g** bearing the 2,3‐dichlorophenyl as RHS, we decided to further investigate the RHS.

**TABLE 1:** SAR investigation of the LHS amine region R.

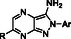			
Entry	R	Ar	SHP2 ‍IC_50_, ‍‍‍nM^a^
**13**	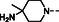	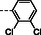	540 ± 25
**17a**	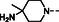	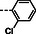	>10,000
**17b**	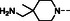	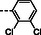	>10,000
**17c**	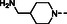	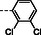	>10,000
**17d**	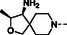	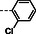	>10,000
**17e**	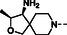	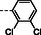	6,100 ± 990
**17f**	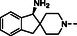	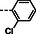	560 ± 56
**17g**	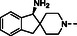	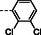	49 ± 4

a The SHP2 enzymatic inhibition (IC_50_) of the compounds was measured under the following conditions: 2 nM WT full‐length SHP2, 2 μM activating peptide, 50 µM DIFMUP, with assay buffer of 60 mM HEPES pH 7.2, 75 mM NaCl, 75 mM KCl, 1 mM EDTA, 5 mM DTT, 0.05% Tween‐20, 0.1% BSA. Data were shown as mean ± SD from three technical replicates. Reference compound values: SHP099 IC_50_ = 150 ± 19 nM and TNO155 IC_50_ = 9 ± 1 nM.

Considering the strong impact of the substitution pattern of the aromatic RHS, we turned our attention to the investigation of various RHS moieties. For instance, an 11‐fold increase in potency between compound **17g** and compound **17f** was observed solely due to the addition of one chlorine in the *meta* position. The importance of the chlorine substitution for SHP2 inhibition was confirmed by the decrease in the biological activity of compound **17h**, lacking both the *ortho* and *meta* chlorine, compared to compounds **17f** and **17g** (Table 2). While **17h** exhibited only a modest (<2‐fold) decrease in potency relative to the mono‐chloro derivative **17f**, a more significant (>20‐fold) reduction in activity was observed compared to **17g** bearing both the *ortho* and *meta* dichloro substitution pattern. Further attempts for activity improvement by incorporating, e.g., a nitrile, an ester, or a carboxyl group in the *meta* and *para* position (compounds **17i–17l**) did not result in higher activity. Overall, the presence of the 2,3‐dichlorophenyl ring as an RHS substituent was deemed optimal for SHP2 inhibition at this stage.

**TABLE 2:** SAR investigation of the RHS aromatic ring Ar.

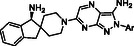		
Entry	Ar	SHP2 IC_50,_ nM^a^
**17h**	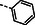	1,100 ± 98
**17i**	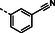	4,200 ± 250
**17j**	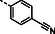	5,300 ± 440
**17k**	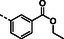	>10,000
**17l**	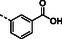	>10,000

a The SHP2 enzymatic inhibition (IC_50_) of the compounds was measured under the following conditions: 2 nM WT full‐length SHP2, 2 μM activating peptide, 50 µM DIFMUP, with assay buffer of 60 mM HEPES pH 7.2, 75 mM NaCl, 75 mM KCl, 1 mM EDTA, 5 mM DTT, 0.05% Tween‐20, 0.1% BSA. Data were shown as mean ± SD from three technical replicates. Reference compound values: SHP099 IC_50_ = 150 ± 19 nM and TNO155 IC_50_ = 9 ± 1 nM.

We next examined the role of the amine group in the 3‐position on the indazole core, which, based on our docking analysis, likely acts as a hydrogen bond donor to the Glu250 backbone carbonyl. As a result, compound **18** showed a fourfold decrease in activity compared to **17f**, emphasizing the importance of this amine group for binding.

Furthermore, to probe the role of the nitrogen atoms present in the amino‐2*H*‐azaindazole scaffold, several analogs with different *N*‐heterocyclic cores were synthesized (Table 3). Notably, replacing the pyrazine ring with a pyrimidine ring in **21a, b,** and eliminating the nitrogen N‐4 in **21c**, or both N‐4 and N‐6 in **21d**, resulted in a significant loss of potency (IC_50_  > 10 µM), rendering compounds **21a–d** inactive and highlighting the importance of the pyrazine moiety for the activity.

**TABLE 3:** Exploration of modification in the 3‐amino‐2*H*‐azaindazole core.

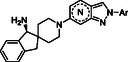			
Entry	Core	Ar	SHP2 IC_50,_ nM^a^
**18**	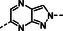	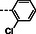	2,500 ± 14
**21a**	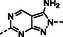	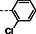	>10,000
**21b**	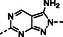	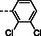	>10,000
**21c**	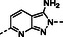	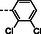	>10,000
**21d**	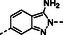	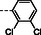	>10,000

a The SHP2 enzymatic inhibition (IC_50_) of the compounds was measured under the following conditions: 2 nM WT full‐length SHP2, 2 μM activating peptide, 50 µM DIFMUP, with assay buffer of 60 mM HEPES pH 7.2, 75 mM NaCl, 75 mM KCl, 1 mM EDTA, 5 mM DTT, 0.05% Tween‐20, 0.1% BSA. Data were shown as mean ± SD from three technical replicates. Reference compound values: SHP099 IC_50_ = 150 ± 19 nM and TNO155 IC_50_ = 9 ± 1 nM.

Overall, compound **17g** demonstrated the most potent inhibition of SHP2 and was therefore selected for subsequent cellular characterization. To validate the allosteric mode of action of compound **17g**, we first assessed its activity against the isolated catalytic PTP domain of SHP2. At 20 µM, the compound displayed only 11% inhibition, supporting the hypothesis that our new inhibitor binds in the allosteric tunnel pocket of SHP2. To obtain structural insight into its binding mode, we next determined the co‐crystal structure of **17g** in complex with SHP2.

### 2.4 Determination of the Binding Mode of 17g in SHP2 by X‐Ray Crystallography

Isothermal titration calorimetry (ITC) was used to determine the binding affinity of compound **17g** to a SHP2 construct harboring both SH2 domains and the PTP domain (SHP2, aa 2–525), revealing a K_D_ value of 780 nM (Figure S2A). The signature plot of the ITC measurement shows that the binding involves many favorable specific interactions such as hydrogen‐bonds, electrostatic interactions, van der Waals contacts, as indicated by an enthalpy of Δ*H* = −39 kJ/mol (Figure S2B). The free enthalpy of Δ*G* = −34 kJ/mol clearly reflects a favorable overall binding with a tight interaction pattern. However, the entropy term −TΔ*S* = 4.5 kJ/mol further underscores the penalty arising from structural conformational change by the C‐SH2 domain movement upon binding of compound **17g**.

**FIGURE 4:**
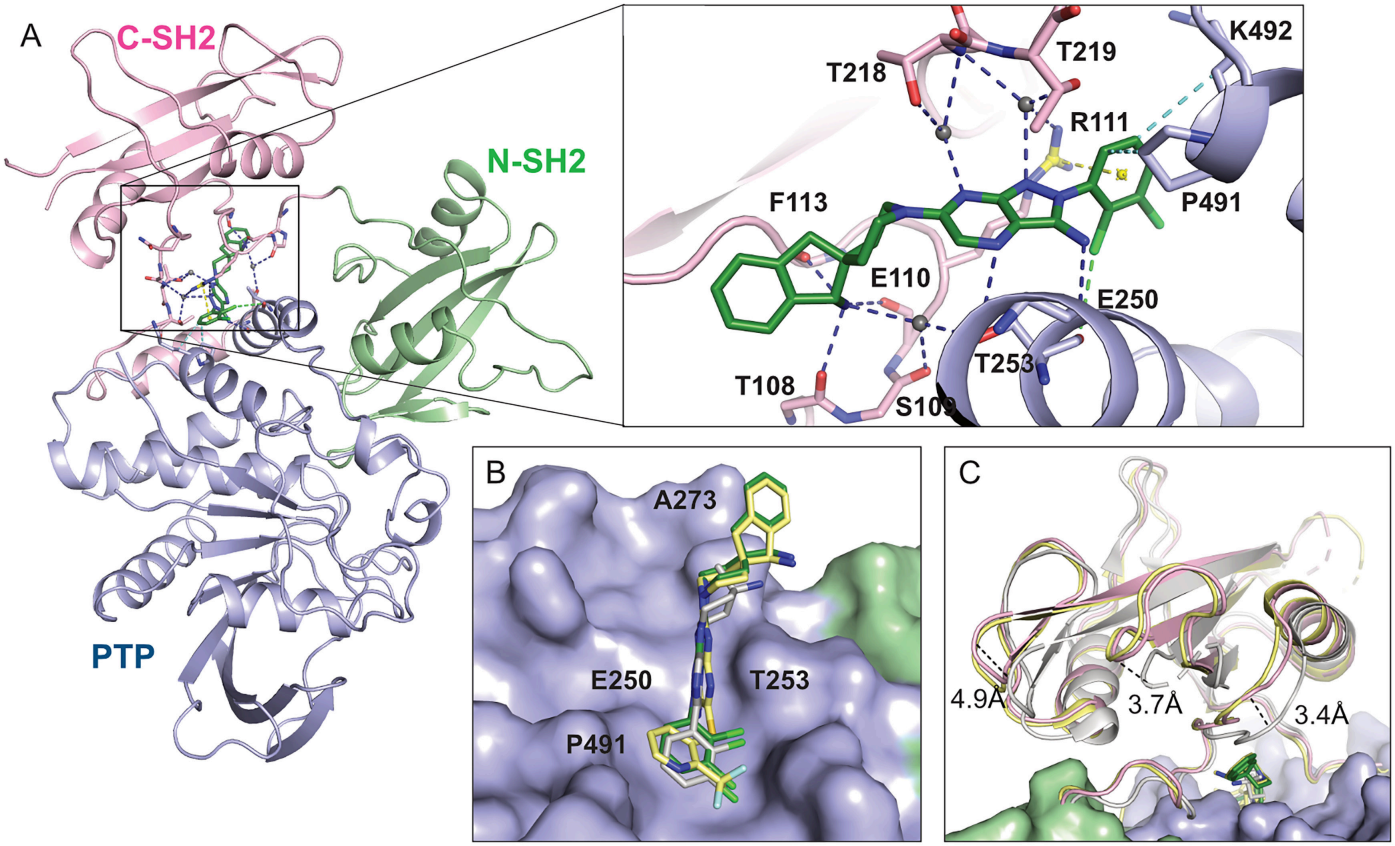
Crystallographic structure analysis of compound **17g** bound to SHP2 allosteric pocket (PBD ID: 9TKU). (A) The SHP2 structure is presented as a cartoon with the N‐terminal SH2 domain in green, the C‐terminal SH2 domain in pink, the PTP domain in blue, and compound **17g** as a stick model in dark green. The magnified inset depicts the detailed binding mode of compound **17g** in *a*  ∼90° left‐rotated and flipped view for better visualization. Observed interactions are shown as dashed lines with H‐bonds highlighted in dark blue, halogen bonds in green, and cation‐π in yellow. (B) Superimposition of compound **17g** (dark green) with SHP099 **1** (light gray) and SHP8 **4** (yellow) in the binding tunnel, with the C‐terminal SH2 domain removed for better illustration. (C) Movement of C‐terminal SH2 domain (pink) upon compound **17g** and SHP8 **4** (PDB ID: 8B5Y, yellow) binding compared to SHP2 (PDB ID: 5EHR) with bound inhibitor SHP099 **1** (light gray). The shift of the SH2 domain is depicted by dashed lines, and the distance is labeled in angstroms between identical residues in the structure.

The SHP2 construct was then cocrystallized with compound **17g** for X‐ray structure determination at 1.9 Å resolution (PBD ID: 9TKU; Figure 4, Table S1). The structure confirmed that **17g** occupies the same allosteric site 1 tunnel‐like SHP2 binding pocket as previously described for other inhibitors, such as SHP099. As shown in the inset of Figure 4A, compound **17g** directly binds to SHP2 with the terminal amine of the spiro‐piperidine headgroup engaged in hydrogen bond interactions with the backbone carbonyls of Thr108, Glu110, Phe113, and water‐mediated hydrogen bonds to Ser109 and Thr253. Moreover, the 3‐amino group of the 2*H*‐azaindazole core acts, as predicted by our docking studies, as a hydrogen bond donor to the backbone carbonyl of Glu250. We also observed a weak hydrogen bond interaction (3.6 Å) between Thr253 and the pyrazine’s nitrogen N‐4, explaining the loss of potency observed for compounds **21c** and **21d**. Furthermore, a direct and a water‐mediated hydrogen bond interaction between the azaindazole nitrogen N‐1 and Arg111, as well as Thr219, was found. The functional importance of these pyrazine‐mediated contacts is directly illustrated by superimposing compound **21d** onto the co‐crystal structure of **17g** bound to SHP2 (PDB ID: 9TKU; Figure S3). The structural overlay reveals the concurrent loss of the N‐4 hydrogen bond to Thr253 and the N‐7 water‐mediated contact to Thr218, which together account for the complete loss of SHP2 inhibitory activity observed for **21d** and further rationalize the potency difference between **17g** and **21c**, in which only N‐4 is absent. Finally, we observed a cation‐π stacking interaction between the RHS 2,3‐dichlorophenyl ring and the Arg111 sidechain, as well as a halogen bond between Thr253 and the *ortho*‐chlorine. Comparison of the novel SHP2‐**17g** co‐crystal structure with previously reported SHP2 complexes revealed a notable structural distinction. Compound **17g** inserts deeper into the tunnel formed between the C‐SH2 and the PTP domains, compared to inhibitors such as SHP099. This deeper insertion forces a displacement of the C‐SH2 domain by up to 4.9 Å away from the PTP domain (Figure 4B,C), hence preventing a steric clash with the SHP2 loop region (amino acids 113−177). As a result, the allosteric binding tunnel is widened, enabling **17g** to interact not only directly with SHP2 but also via various water‐mediated contacts, which were not observed previously for compounds bound to the allosteric binding tunnel.

A similar structural rearrangement has been reported for the co‐crystal structure of SHP2 with compound **4 (**PDB ID: 8B5Y) [14]. Superimposition with compound **17g** revealed a nearly identical positioning of the spiroamine moiety within the tunnel and a comparable displacement of the C‐SH2 domain (Figure 4B,C). Nevertheless, compound **4** exhibits higher biochemical potency (IC_50_ = 5 nM) than compound **17g** (IC_50_ = 49 nM). Inspection of the co‐crystal structure of SHP2 in complex with compound **4** reveals an interaction network centered on Arg111 and Glu249, including a direct hydrogen bond to Glu249 and extensive stabilization of Arg111 through hydrogen bonds, cation‐π interactions, and water‐mediated interactions [14]. However, while **17g** also engages with Arg111 and induces a similar domain movement, the absence of a direct Glu249 interaction and the resulting subtle differences in the water–mediated interactions could explain the observed difference in inhibitory potency.

### 2.5 Cell‐Based Evaluation

Given the notable enzymatic activity of the novel 3‐amino‐2*H*‐azaindazole allosteric SHP2 inhibitor, we next evaluated the aqueous solubility of compound **17g** to assess its suitability for further cellular profiling. Kinetic solubility measured in PBS buffer was 190 µM, a value in the same range as that of SHP099 **1** (230 µM), suggesting a good solubility compatible with cellular applications (Table 4).

**TABLE 4:** Pharmacological evaluation of **17g**.

Entry	**17g** (3‐amino‐2*H*‐‍azaindazole)	1 (SHP099)	2 (TNO155)
Kinetic solubility (µM)^a^	190	230	n.d.
Growth Inhibition IC_50_ (MV‐4‐11 cells, nM)^b^	70 ± 30	270 ± 92	6.0 ± 2.0
SHP2^E76K^ IC_50_ (MV‐4‐11 cells, nM)^c^	8,500 ± 300	20,000 ± 800	500 ± 20
IC_50_ pERK (MV‐4‐11 cells, nM)^d^	50 ± 7	n.d.	8 ± 4
IC_50_ pERK (Panc‐1 cells, nM)^d^	250 ± 61	430 ± 72	46 ± 15
IC_50_ pERK (KYSE520 cells, nM)^d^	410 ± 133	900 ± 133	88 ± 27

a Kinetic solubility determined in PBS (pH = 7.4) after 24h incubation at room temperature. The reported values are the mean of duplicate measurements.

b,c MV‐4‐11 or MV‐4‐11 overexpressing SHP2^E76K^ (10000 cells/well) were plated onto 96‐well plates and treated with test compounds. After 72 h, cell viability was quantified using a CCK‐8 assay. IC_50_ values are reported as mean ± SD from independent experiments.

d MV‐4‐11 cells, Panc‐1 cells, and KYSE520 cells were treated with test compounds for 1h, and pERK levels were quantified using an in‐cell Western Blot assay. IC_50_ values are reported as mean ± SD from three independent experiments. N.d. not determined.

Next, we measured the anti‐proliferative activity of compound **17g** in a cell growth inhibition assay using the acute myeloid leukemia (AML) cell line MV‐4–11, where aberrant SHP2 signaling contributes to oncogenic transformation [59, 60]**,** with SHP099 **1** and TNO155 **2** serving as positive controls. In this model, compound **17g** displayed an IC_50_ of 70 nM, outperforming SHP099 **1** (IC_50_ = 270 nM), and demonstrating a level of activity that supports further optimization. As expected, TNO155 **2** was the most potent of the three, with an antiproliferative IC_50_ of 6 nM.

We also examined the ability of **17g** to inhibit oncogenic SHP2 mutants, focusing on the clinically relevant E76K mutation, which is known to destabilize the autoinhibited closed conformation and confer resistance to allosteric inhibitors [61, 62, 63]. Using MV‐4‐11 cells overexpressing SHP2^E76K^, we found that compound **17g** retained measurable activity, although reduced with an IC_50_ of 8.5 µM, showing a clear improvement over SHP099 (IC_50_ = 20 µM) but significantly less potent than TNO155 (IC_50_ = 0.5 µM) (Table 4).

SHP2 acts as a key regulator of cell survival and proliferation, primarily through its role in MAPK pathway activation downstream of receptor tyrosine kinases [9]. Hence, to complement the antiproliferative studies performed in MV‐4‐11 cells and to directly assess pathway modulation, we evaluated the effect of compound **17g** on ERK phosphorylation in this cell line. Compound **17g** inhibited pERK with an IC_50_ value of 50 nM, in close agreement with its antiproliferative IC_50_ of 70 nM in the same MV‐4‐11 cell line. This provides direct evidence that the observed growth inhibition is driven by suppression of the SHP2‐ mediated MAPK signaling. Here, TNO155 **2** again served as a benchmark, inhibiting pERK in MV‐4‐11 cells with an IC_50_ of 8 nM, consistent with its superior antiproliferative potency.

To further characterize the cellular potency of compound **17g**, we next evaluated the influence of compound **17g** on the phosphorylation level of ERK in Panc‐1 pancreatic cancer and KYSE520 esophageal squamous carcinoma cell lines, as both rely on SHP2‐mediated MAPK signaling. In these models, compound **17g** inhibited ERK phosphorylation with cellular IC_50_ values of 250 nM and 410 nM, respectively. While the clinical benchmark TNO155 **2** with IC_50_ values of 46 nM and 88 nM, respectively remained more potent, compound **17g** demonstrates clear improvement over the first‐generation allosteric inhibitor SHP099 **1** (IC_50_ = 430 and 900 nM, respectively) in both cellular models. These findings confirm that compound **17g** is sufficiently cell‐permeable and capable of modulating SHP2‐mediated ERK signaling in a cellular context.

## 3 Conclusion

In this study, we report the discovery of a new class of allosteric SHP2 inhibitors with a 3‐amino‐2*H*‐azaindazole as a scaffold. SAR investigations revealed that the presence of a 2,3‐dichlorophenyl group on the RHS and a spiro‐amine head group on the LHS are critical for maintaining potent SHP2 inhibition. Furthermore, our work introduces a new geometry of 150° between the LHS and RHS pharmacophores within the SHP2 allosteric pocket. This new spatial arrangement underscores the flexibility of the SHP2 allosteric binding site and provides a strong rationale for further exploration of nonconventional geometries in the design of future inhibitors.

The diverse analogs synthesized using our previously developed, highly regioselective approach to 3‐amino‐2*H*‐indazoles highlight the robustness of this synthesis strategy, while further reinforcing the emerging value of the 2*H*‐indazole scaffold in medicinal chemistry. Among these compounds, **17g** was the most potent SHP2 allosteric inhibitor with an enzymatic inhibitory activity IC_50_ = 49 nM. In cellular assays, compound **17g** also displayed good cellular activity by inhibiting the phosphorylation of ERK in MV‐4‐11, Panc‐1, and KYSE520 cell lines.

Structural investigation of the binding mode of compound **17g** revealed a rearrangement of the C‐SH2 domain after insertion of the inhibitor into the allosteric binding tunnel to minimize steric hindrance. As a result, the binding site expands, enabling compound **17g** to engage in an extensive hydrogen bond network, which also involves water molecules. Such a binding mode has been rarely reported for SHP2 allosteric inhibitors.

Altogether, these results position compound **17g** as a promising lead for further optimization and development and validate our scaffold‐ and geometry‐based approach to design novel allosteric SHP2 inhibitors. More broadly, this work expands the structural landscape of SHP2 allosteric inhibition and provides a framework for the rational design of new inhibitors with distinct binding modes and improved pharmacological profiles.

## Funding

This study was supported by Deutsche Forschungsgemeinschaft (Grant 456689823), and European Union’s Horizon 2020 research and innovation program (Grant 956314).

## Conflicts of Interest

The authors declare no conflicts of interest.

## Supporting information

Supplementary Material

## Data Availability

The data that supports the findings of this study are available in the supplementary material of this article.
