# Supplementary material for: Probing 3‐Amino‐2H‐Azaindazoles as Allosteric Inhibitors of the Protein Tyrosine Phosphatase SHP2
Source: ChemMedChem. 2026 Jun 14;21(11):e70341. doi: 10.1002/cmdc.70341 (PMC13265398; doi:10.1002/cmdc.70341)
Supplement: Supplementary file 1 — Supplementary Material [file CMDC-21-e70341-s001.pdf]

Supporting Information

Probing 3-Amino-2*H*-Azaindazoles as Allosteric Inhibitors of the Protein Tyrosine Phosphatase SHP2

Machoud Amoussa,#[a,b] Nina-Louisa Efrém,#[a,c] Feng Li,#[d,e] Ziqiong Guo,[a,b] Yvette Roske,[f] Katrin Jana Frank,[g] Szymon Pach,[b,h] Clemens Alexander Wolf,[b] Feng Bo,[e] Marina Lesina,[g] Mika Kintzel,[a] Rana Alsalim,[a] Victoria Zeitz,[a] Noémi Csorba,[i] Silke Radetzki,[a] György M. Keserű,[i] Oliver Daumke,[f] Hana Algül,[g] Gerhard Wolber,[b] Jia Li,*[d,e] Marc Nazaré*[a,j]

---

[a] M. Amoussa, N.-L. Efrém, Z. Guo, M. Kintzel, R. Alsalim, V. Zeitz, S. Radetzki, M. Nazaré
Leibniz-Forschungsinstitut für Molekulare Pharmakologie (FMP), Medicinal Chemistry
Campus Berlin-Buch, Robert-Rössle-Str. 10, 13125 Berlin, Germany.
[b] M. Amoussa, Z. Guo, S. Pach, C. A. Wolf, G. Wolber
Freie Universität, Molecular Design Group, Institute of Pharmacy
Königin-Luise-Str. 2–4, 14195 Berlin, Germany.
[c] N.-L. Efrém
Karlsruhe Institute of Technology, Institute of Organic Chemistry, Organic Chemistry I
Fritz-Haber-Weg 6, 76131 Karlsruhe, Germany.
[d] F. Li, J. Li
School of Chinese Materia Medica, Nanjing University of Chinese Medicine, Nanjing, China
[e] F. Li, F. Bo, J. Li
State Key Laboratory of Chemical Biology, Shanghai Institute of Materia Medica (SIMM), Chinese Academy of Sciences
555 Zuchongzhi Road, Zhang Jiang Hi-Tech Park, Pudong, Shanghai, China.
[f] Y. Roske, O. Daumke
Max Delbrück Center for Molecular Medicine (MDC), Structural Biology
Campus Berlin-Buch, Robert-Rössle-Str. 10, 13125 Berlin, Germany.
[g] K. J. Frank, M. Lesina, H. Algül
Comprehensive Cancer Center München, Institute for Tumor Metabolism, Klinikum rechts der Isar, Technical University of Munich, School of Medicine and Health, Ismaninger Str. 22, 81675 Munich, Bavaria, Germany.
[h] S. Pach
Philipps-Universität Marburg, Department of Pharmaceutical Chemistry
Marbacher Weg 8, Marburg 35037, Germany.
[i] N. Csorba, G. M. Keserű
Research Centre for Natural Sciences (RCNS)
Magyar tudósok körútja 2, 1117 Budapest, Hungary.
National Laboratory for Drug Research and Development, Magyar tudósok krt. 2, H-1117 Budapest, Hungary.
Department of Organic Chemistry and Technology, Faculty of Chemical Technology and Biotechnology, Budapest University of Technology and Economics, Műegyetem rkp. 3, H-1111 Budapest, Hungary.
[j] M. Nazaré
Bielefeld University, Department of Chemistry, Organic and Bioorganic Chemistry
Universitätsstraße 25, 33615 Bielefeld, Germany.

*E-mail: jli@simm.ac.cn, nazare@fmp-berlin.de

Table of Contents

Chemistry.......... S3

Materials and Methods S3

General procedure 1: Formation of the halo-benzonitrile derivatives (scale: 0.5 - 1.0 mmol).... S4

General procedure 2: Formation of the 3-amino-2*H*-azaindazoles (scale: 0.1 - 0.25 mmol) [1].. S4

Biochemical SHP2 assay S19

Growth inhibition MV-4-11 cells S19

Construction of SHP2 mutated cells.......... S19

pERK cellular assay MV-4-11 cells.......... S20

pERK cellular assay Panc-1 and KYSE520 cells.......... S20

SHP2 overexpression, purification S20

Crystallization and structure determination.......... S21

Molecular docking studies.......... S22

Isothermal titration calorimetry (ITC) S22

Measurement of the kinetic solubility.......... S23

NMR spectra (^{1}H-NMR and ^{13}C-NMR) spectra of key compounds and intermediates S24

Supplementary Table S1: Data collection and refinement statistics.......... S33

Superimposition of compound 21d onto compound 17g.......... S34

Reference S34

Chemistry

Materials and Methods

Chemicals and anhydrous solvents were purchased from commercial suppliers (Sigma-Aldrich, BLDPharm, Abcr, Carbolution, TCI) and used as received unless otherwise specified. **TLC** was carried out on aluminum-backed silica gel plates (silica gel 60 F 254, Merck), visualizing with UV light (λ = 254 nm). **LC-MS** was performed with an Agilent 1260 series HPLC system employing a DAD detector (at 600, 500, 300, 254, and 220 nm) equipped with Agilent Technologies 6120 Quadrupole LC/MS in electrospray positive mode (ESI+). A Thermo Accuore RP-MS (30 × 2.1 mm, 2.6 µm) column was used with a flow rate 0.8 mL/min in combination with the following separation conditions: 0.1% formic acid in water (solvent A); 0.1% formic acid in ACN (solvent B); 5% B for 0.2 min, from 5 to 95% B in 0.9 min, 95% B for 1.4 min (stop point at 2.5 min). Data analysis was performed with ChemStation software. **Purification** via flash chromatography was carried out using Biotage Isolera One apparatus or Combiflash NextGen 300+ apparatus equipped with an ELSD detector using RediSep®Rf columns from Teledyne Isco, using gradients of ethyl acetate in cyclohexane (0% to 100%). Preparative reversed-phase HPLC was performed on a Gilson PLC 2250 with a Macherey-Nagel VP 250/21 Nucleodur 100-7 C18Ec column (30 mL/min flow) or a Macherey-Nagel VP 250/10 Nucleodur 100-5 C18Ec column (5 mL/min flow). Elution was performed using solvent A (H_2O containing 0.1% formic acid or trifluoroacetic acid) and solvent B (acetonitrile containing 0.1% formic acid or trifluoroacetic acid) with a gradient from 5 to 95% solvent B over 45min. **HRMS** analyses were carried out on Agilent Technologies 6530 Accurate Mass Q-ToF LC/MS linked to Agilent Technologies HPLC 1260 Infinity II. **NMR** spectra were recorded at either Bruker AV 300 (295K, 300 MHz, 75 MHz) or Bruker AV 600 (300K, 600 MHz, 151 MHz) spectrometers in $CDCl_3$, $C\mathit{D}_3O\mathit{D}$ and DMSO-d_6 as solvents. All ^{13}C NMR spectra were recorded with ^{1}H NMR-broad-band decoupling. All chemical shifts are reported in ppm (δ) relative to the residual solvent signals of $CDCl_3$, $C\mathit{D}_3O\mathit{D}$, and DMSO-d_6 (δ = ^{1}H NMR: 7.26, 3.31, and 2.50 ppm; ^{13}C NMR: 77.16, 49.00, and 39.5 ppm, respectively). NMR data were analyzed with MestReNova 14.2.1 software. **Compound names** were generated with ChemDraw 20.1 software and are not necessarily identical to IUPAC nomenclature. Purity and characterization of all final compounds were established by a combination of LC-MS, LC-HRMS, and NMR analytical techniques. All tested compounds were found to be >95% pure by LC/MS and HRMS analysis.

General procedure 1: Formation of the halo-benzonitrile derivatives (scale: 0.5 - 1.0 mmol)

To a solution of halo-benzonitrile (1 eq.) in anhydrous DCM (0.2 M) were added the amine derivative (1.5 eq.) and DIPEA (3 eq.). The mixture was stirred at room temperature for 3h. When applicable, Boc_2O (2 eq.) was added and left stirred at room temperature overnight. The mixture was then diluted with water (30 mL) and extracted three times with DCM (3 x 20 mL). The organic phase was dried over $MgSO_4$, filtered, and concentrated under reduced pressure. The obtained crude mixture was then purified by column chromatography (SiO_2, 0% to 100% ethyl acetate in cyclohexane) to yield the desired halo-benzonitrile derivatives **15a-15e** and **20a-20b**.

General procedure 2: Formation of the 3-amino-2*H*-azaindazoles (scale: 0.1 - 0.25 mmol) [1]

In an oven-dried microwave tube, $Pd_2(dba)_3$ (20 mol%), tBu_3PHBF_4 (30 mol%), Cs_2CO_3 (3 eq.), and anhydrous 1,4-dioxane (0.1 M) were added. The resulting mixture was stirred under nitrogen atmosphere at room temperature for 30 min. Then the corresponding halo-benzonitrile derivative from general procedure 1 (1 eq.) and the respective hydrazine (2 eq.) were added to the reaction mixture and heated in a block heating system to 112°C for 2-4h. After cooling to room temperature, the reaction mixture was filtered over Celite with DCM. The filtrate was then poured into a separatory funnel containing water (15 mL) and was extracted three times with DCM (3 × 15 mL). The combined organic phases were dried over $MgSO_4$ and concentrated under reduced pressure. When applicable, the obtained Boc-protected crude intermediate was deprotected by stirring in 20% TFA in DCM (v/v, 10 mL per mmol of substrate) for 2h. The solution was co-evaporated with toluene twice to give the crude primary amine TFA salt. The obtained crude product was then purified by reversed-phase preparative HPLC (C18 column, solvent A: H_2O/0.1% FA or TFA, solvent B: ACN/0.1% FA or TFA. Gradient 5-95% solvent B over 45 min) to achieve the desired 3-amino-2*H*-azaindazole derivatives **13, 17a-17l, 18,** and **21a-21d.**

***tert*-Butyl (1-(6-chloro-5-cyanopyrazin-2-yl)-4-methylpiperidin-4-yl)carbamate (15a)**

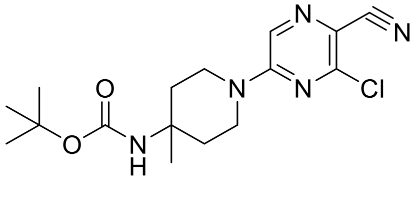

The title compound was synthesized following general procedure 1 using 3,5-dichloropyrazine-2-carbonitrile (87 mg, 0.50 mmol, 1.00 eq.) and tert-Butyl (4-methylpiperidin-4-yl)carbamate (160

mg, 0.75 mmol, 1.50 eq.). The desired product was obtained as a yellow oil (148 mg, 85%). LC-MS (ESI) (m/z) $[M+H]^+$= 352.1; HRMS (ESI) (m/z): calcd for $C_{16}H_{22}ClN_5O_2$ $[M+H]^+$ 352.1703, found 352.1700; ^{1}H NMR (300 MHz, $CDCl_3$) δ 8.03 (s, 1H), 4.45 (s, 1H), 4.04 (d, J = 13.5 Hz, 2H), 3.49 (ddd, J = 13.9, 10.7, 3.3 Hz, 2H), 2.32 – 2.16 (m, 2H), 1.65 (ddd, J = 14.5, 10.7, 4.3 Hz, 2H), 1.46 (s, 9H), 1.42 (s, 3H) ppm. ^{13}C NMR (75 MHz, $CDCl_3$) δ 171.20, 153.08, 150.32, 128.53, 115.71, 113.83, 79.69, 77.25, 60.43, 50.44, 40.89, 35.76, 28.41, 26.30, 21.09, 14.22 ppm.

***tert*-Butyl ((1-(6-chloro-5-cyanopyrazin-2-yl)-4-methylpiperidin-4-yl)methyl)carbamate (15b)**

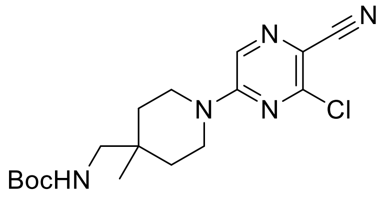

The title compound was synthesized following general procedure 1 using 3,5-dichloropyrazine-2-carbonitrile (87 mg, 0.50 mmol, 1.00 eq.) and tert-Butyl N-[(4-methylpiperidin-4-yl)methyl]carbamate (171 mg, 0.75 mmol, 1.50 eq.). The desired product was obtained as a pale yellow oil (85 mg, 46%). LC-MS (ESI) (m/z) $[M+H]^+$= 366.1; HRMS (ESI) (m/z): calcd for $C_{17}H_{24}ClN_5O_2$ $[M+H]^+$ 366.1691, found 366.1660; ^{1}H NMR (300 MHz, MeOD) δ 8.20 (s, 1H), 4.01 (dt, J = 11.3, 4.6 Hz, 2H), 3.62 (ddd, J = 13.4, 9.1, 3.8 Hz, 2H), 3.08 – 2.99 (m, 2H), 1.63 – 1.47 (m, 4H), 1.46 (s, 9H), 1.04 (s, 3H) ppm. ^{13}C NMR (75 MHz, MeOD) δ 157.49, 153.32, 149.81, 129.64, 115.61, 111.83, 78.57, 48.97, 40.69, 33.63, 33.36, 27.43, 21.38 ppm.

***tert*-Butyl ((1-(6-chloro-5-cyanopyrazin-2-yl)piperidin-4-yl)methyl)carbamate (15c)**

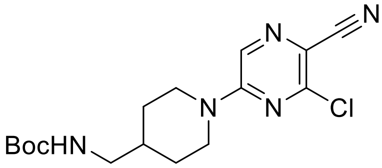

The title compound was synthesized following general procedure 1 using 3,5-dichloropyrazine-2-carbonitrile (87 mg, 0.50 mmol, 1.00 eq.) and tert-Butyl (piperidin-4-ylmethyl)carbamate (160 mg, 0.75 mmol, 1.50 eq.). The desired product was obtained as a yellow oil, which was used as a crude product without further purification (120 mg, 68%). LC-MS (ESI) (m/z) $[M+H]^+$= 352.1; HRMS (ESI) (m/z): calcd for $C_{16}H_{22}ClN_5O_2$ $[M+H]^+$ 352.1535, found 352.1526; ^{1}H NMR (300 MHz, MeOD) δ 8.22 (s, 1H), 6.77 (t, J = 6.2 Hz, 1H), 4.54 (d, J = 13.3 Hz, 2H), 3.18 – 2.92 (m, 4H),

1.97 – 1.75 (m, 3H), 1.46 (s, 9H), 1.33 – 1.11 (m, 2H) ppm. ^{13}C NMR (75 MHz, MeOD) δ 157.27, 153.31, 149.81, 129.68, 115.55, 111.93, 78.59, 45.08, 44.40, 36.41, 29.11, 27.38 ppm.

***tert*-Butyl (S)-(1'-(6-chloro-5-cyanopyrazin-2-yl)-1,3-dihydrospiro[indene-2,4'-piperidin]-1-yl)carbamate (15d)**

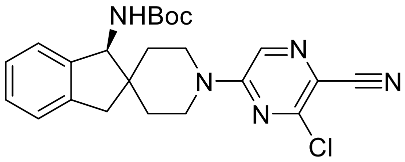

The title compound was synthesized following general procedure 1 using 3,5-dichloropyrazine-2-carbonitrile (174 mg, 1.00 mmol, 1.00 eq.) and (1S)-1,3-dihydrospiro[indene-2,4'-piperidin]-1-amine dihydrochloride (413 mg, 1.50 mmol, 1.50 eq.). The desired product was obtained as a yellow oil (365 mg, 83%). LC-MS (ESI) (m/z) $[M+H]^+$= 440.1; HRMS (ESI) (m/z): calcd for $C_{23}H_{26}ClN_5O_2$ $[M+H]^+$ 440.1848, found 440.1860; ^1H NMR (300 MHz, $CDCl_3$) δ 8.03 (s, 1H), 7.29 (m, 4H), 5.04 (d, J = 9.5 Hz, 1H), 4.66 (d, J = 9.6 Hz, 1H), 4.22 (s, 2H), 3.47 (td, J = 12.1, 3.9 Hz, 2H), 3.06 (d, J = 15.9 Hz, 1H), 2.84 (d, J = 15.9 Hz, 1H), 2.04 – 1.67 (m, 4H), 1.49 (s, 9H) ppm. ^{13}C NMR (75 MHz, $CDCl_3$) δ 155.85, 153.05, 150.38, 141.54, 140.39, 128.54, 128.50, 127.35, 125.26, 124.76, 115.80, 113.60, 80.07, 62.91, 46.17, 42.40, 41.86, 40.59, 35.22, 29.79, 28.38 ppm.

***tert*-Butyl ((3S,4S)-8-(6-chloro-5-cyanopyrazin-2-yl)-3-methyl-2-oxa-8-azaspiro[4.5]decan-4-yl)carbamate (15e)**

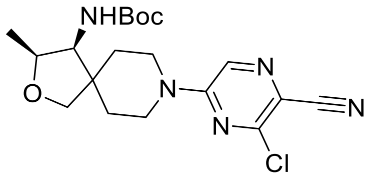

The title compound was synthesized following general procedure 1 using 3,5-dichloropyrazine-2-carbonitrile (87 mg, 0.50 mmol, 1.00 eq.) and (3S,4S)-3-Methyl-2-oxa-8-azaspiro[4.5]decan-4-amine dihydrochloride (206 mg, 0.75 mmol, 1.50 eq.). The desired product was obtained as a yellow oil (153 mg, 75%). LC-MS (ESI) (m/z) $[M+H]^+$= 408.1; HRMS (ESI) (m/z): calcd for $C_{19}H_{26}ClN_5O_3$ $[M+H]^+$ 408.1797, found 408.1783; ^1H NMR (300 MHz, MeOD) δ 8.24 (s, 1H), 7.11 – 6.91 (m, 1H), 4.27 (pd, J = 7.3, 5.5 Hz, 1H), 4.01 (dd, J = 10.6, 4.7 Hz, 1H), 3.89 (t, J = 5.8 Hz, 2H), 3.79 – 3.70 (m, 2H), 3.70 – 3.58 (m, 1H), 1.94 – 1.55 (m, 4H), 1.47 (s, 9H), 1.17 (d, J = 6.3 Hz, 3H) ppm. ^{13}C NMR (75 MHz, MeOD) δ 157.23, 157.17, 153.33, 149.80, 129.74, 115.51,

112.17, 79.04, 78.94, 76.20, 76.11, 75.49, 59.57, 59.48, 45.43, 34.45, 29.45, 27.37, 27.28, 14.08 ppm.

6-(4-Amino-4-methylpiperidin-1-yl)-2-(2,3-dichlorophenyl)-2H-pyrazolo[3,4-b]pyrazin-3-amine (13)

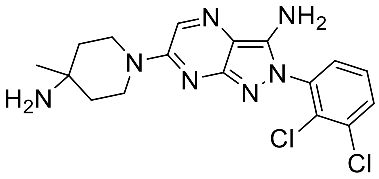

The title compound was synthesized following general procedure 2 using **15a** (70 mg, 0.20 mmol, 1.00 eq.) and 2,3-dichlorophenylhydrazine hydrochloride (72 mg, 0.40 mmol, 2.00 eq.). The desired product was obtained as a yellow amorphous powder (11 mg, 11%). LC-MS (ESI) (m/z) $[M+H]^+$ = 392.1; HRMS (ESI) (m/z): calcd for $C_{17}H_{19}Cl_2N_7$ $[M+H]^+$ 392.1152, found 392.1145; ^1H NMR (300 MHz, MeOD) δ 8.17 (s, 1H), 7.85 – 7.72 (m, 1H), 7.61 – 7.47 (m, 2H), 4.28 (d, *J* = 14.3 Hz, 2H), 3.60 – 3.45 (m, 2H), 1.88 (d, *J* = 5.8 Hz, 4H), 1.51 (s, 3H) ppm. ^{13}C NMR (75 MHz, MeOD) δ 154.86, 151.04, 143.08, 136.93, 133.86, 131.80, 131.57, 129.82, 129.26, 128.56, 128.25, 110.66, 52.02, 40.79, 35.11, 34.76, 21.60 ppm.

6-(4-Amino-4-methylpiperidin-1-yl)-2-(2-chlorophenyl)-2H-pyrazolo[3,4-b]pyrazin-3-amine (17a)

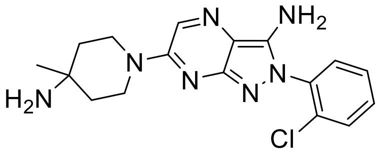

The title compound was synthesized following general procedure 2 using **15a** (95 mg, 0.27 mmol, 1.00 eq.) and 2-chlorophenylhydrazine hydrochloride (96 mg, 0.54 mmol, 2.00 eq.). The desired product was obtained as a yellow amorphous powder (47 mg, 36%). LC-MS (ESI) (m/z) $[M+H]^+$= 358.1; HRMS (ESI) (m/z): calcd for $C_{17}H_{20}ClN_7$ $[M+H]^+$ 358.1541, found 358.1568; ^1H NMR (300 MHz, DMSO-d_6) δ 8.33 (s, 1H), 8.11 (s, 3H), 7.75 (dd, *J* = 7.8, 1.7 Hz, 1H), 7.69 – 7.54 (m, 3H), 4.19 (d, *J* = 14.3 Hz, 2H), 3.57 – 3.41 (m, 2H), 1.79 (q, *J* = 4.3 Hz, 4H), 1.41 (s, 3H) ppm. ^{13}C NMR (75 MHz, DMSO-d_6) δ 158.93, 158.48, 154.97, 149.82, 145.21, 134.08, 132.39, 131.26, 131.01, 130.61, 129.01, 111.21, 52.48, 41.13, 34.70, 22.73 ppm.

6-(4-(Aminomethyl)-4-methylpiperidin-1-yl)-2-(2,3-dichlorophenyl)-2H-pyrazolo[3,4-b]pyrazin-3-amine (17b)

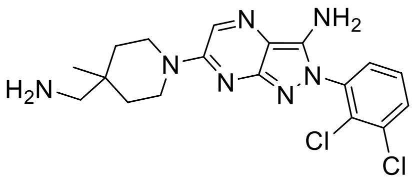

The tile compound was synthesized following general procedure 2 using **15b** (91 mg, 0.25 mmol, 1.00 eq.) and 2,3-dichlorophenylhydrazine hydrochloride (106 mg, 0.50 mmol, 2.00 eq.). The desired product was obtained as a yellow amorphous amorphous powder (14 mg, 13%); LC-MS (ESI) (m/z) $[M+H]^+$= 407.1; HRMS (ESI) (m/z): calcd for $C_{18}H_{21}Cl_2N_7$ $[M+H]^+$ 406.1308, found 406.1298; ^{1}H NMR (300 MHz, DMSO-d_6) δ 8.13 (s, 1H), 7.83 (dd, *J* = 7.2, 2.5 Hz, 1H), 7.59 – 7.49 (m, 2H), 6.54 (s, 1H), 3.97 (dd, *J* = 14.0, 4.9 Hz, 4H), 2.71 (s, 2H), 1.60 – 1.32 (m, 4H), 1.06 (s, 3H) ppm. ^{13}C NMR (75 MHz, DMSO-d_6) δ 154.71, 150.86, 143.09, 138.15, 133.07, 131.74, 131.06, 129.63, 129.37, 129.18, 111.16, 49.34, 40.93, 33.87, 32.39, 29.00, 21.76 ppm.

6-(4-(Aminomethyl)piperidin-1-yl)-2-(2,3-dichlorophenyl)-2H-pyrazolo[3,4-b]pyrazin-3-amine (17c)

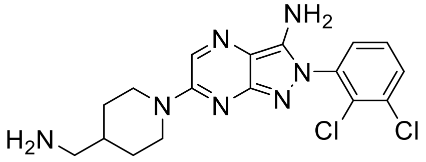

The tile compound was synthesized following general procedure 2 using **15c** (88 mg, 0.25 mmol, 1.00 eq.) and 2-chlorophenylhydrazine hydrochloride (106 mg, 0.50 mmol, 2.00 eq.). The desired product was obtained as a yellow amorphous powder (9 mg, 9%); LC-MS (ESI) (m/z) $[M+H]^+$= 392.1; HRMS (ESI) (m/z): calcd for $C_{17}H_{19}Cl_2N_7$ $[M+H]^+$ 392.1152, found 392.1170; ^{1}H NMR (600 MHz, MeOD) δ 8.55 (s, 1H), 8.14 (s, 1H), 7.78 (dt, J = 8.4, 4.2 Hz, 1H), 7.58 – 7.48 (m, 1H), 4.71 – 4.55 (m, 2H), 3.07 (t, J = 13.1 Hz, 2H), 2.90 (d, J = 7.1 Hz, 2H), 2.68 (s, 1H), 2.08 – 1.85 (m, 4H) ppm. ^{13}C NMR (151 MHz, MeOD) δ 155.00, 151.25, 136.98, 133.86, 131.75, 131.60, 129.72, 129.49, 128.56, 128.20, 44.46, 44.26, 39.04, 34.71, 28.73, 27.46 ppm.

(3S,4S)-8-(3-Amino-2-(2-chlorophenyl)-2H-pyrazolo[3,4-b]pyrazin-6-yl)-3-methyl-2-oxa-8-azaspiro[4.5]decan-4-amine (17d)

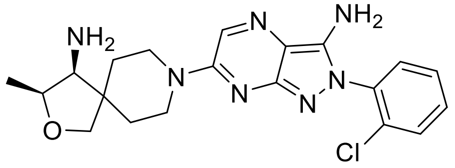

The title compound was synthesized following general procedure 2 using **15e** (101 mg, 0.23 mmol, 1.00 eq.) and 2-chlorophenylhydrazine hydrochloride (82 mg, 0.46 mmol, 2.00 eq.). The desired product was obtained as a yellow amorphous powder (10 mg, 9.8%). LC-MS (ESI) (m/z) $[M+H]^+$= 414.1; HRMS (ESI) (m/z): calcd for $C_{20}H_{24}ClN_7O$ $[M+H]^+$ 414.1804, found 414.1802; ^{1}H NMR (600 MHz, MeOD) δ 7.57 (d, *J* = 3.9 Hz, 1H), 7.31 (d, *J* = 7.9 Hz, 1H), 7.15 (q, *J* = 6.0 Hz, 1H), 6.92 (d, *J* = 8.0 Hz, 1H), 6.83 (t, *J* = 7.5 Hz, 1H), 4.33 – 4.14 (m, 2H), 3.93 (d, *J* = 9.2 Hz, 1H), 3.85 – 3.76 (m, 1H), 3.56 – 3.35 (m, 2H), 3.19 (t, *J* = 11.6 Hz, 2H), 2.04 – 1.60 (m, 4H), 1.32 (d, *J* = 6.2 Hz, 3H) ppm. ^{13}C NMR (75 MHz, MeOD) δ 154.95, 150.96, 135.16, 132.48, 131.20, 130.38, 129.97, 129.24, 127.97, 74.82, 74.10, 61.69, 50.45, 45.10, 42.29, 41.91, 34.60, 29.39, 27.45, 22.22, 13.50 ppm.

(3S,4S)-8-(3-Amino-2-(2,3-dichlorophenyl)-2H-pyrazolo[3,4-b]pyrazin-6-yl)-3-methyl-2-oxa-8-azaspiro[4.5]decan-4-amine (17e)

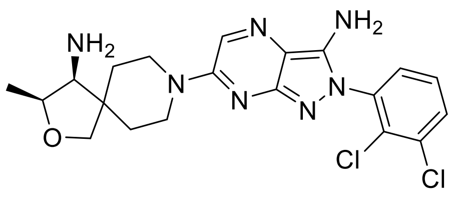

The tile compound was synthesized following general procedure 2 using **15e** (100 mg, 0.25 mmol, 1.00 eq.) and 2,3-dichlorophenylhydrazine hydrochloride (106 mg, 0.50 mmol, 2.00 eq.). The desired product was obtained as a yellow amorphous powder (12 mg, 11%). LC-MS (ESI) (m/z) $[M+H]^+$= 449.1; HRMS (ESI) (m/z): calcd for $C_{20}H_{23}Cl_2N_7O$ $[M+H]^+$ 448.1414, found 448.1404; ^{1}H NMR (300 MHz, MeOD) δ 8.14 (s, 1H), 7.78 (dd, *J* = 6.5, 3.2 Hz, 1H), 7.54 (q, *J* = 4.3 Hz, 2H), 4.37 – 4.18 (m, 3H), 3.95 (d, *J* = 8.9 Hz, 1H), 3.81 (d, *J* = 8.9 Hz, 1H), 3.66 (d, *J* = 2.2 Hz, 1H), 3.47 – 3.37 (m, 1H), 3.21 (d, *J* = 4.5 Hz, 1H), 1.90 – 1.66 (m, 4H), 1.28 (d, *J* = 6.4 Hz, 3H) ppm. ^{13}C NMR (75 MHz, MeOD) δ 155.00, 151.24, 142.96, 136.97, 133.83, 131.74, 131.58, 129.47, 128.59, 128.23, 110.37, 75.88, 74.07, 61.26, 45.29, 42.48, 42.00, 34.44, 29.20, 27.46, 13.90 ppm.

(S)-1'-(3-Amino-2-(2-chlorophenyl)-2H-pyrazolo[3,4-b]pyrazin-6-yl)-1,3-dihydrospiro[indene-2,4'-piperidin]-1-amine (17f)

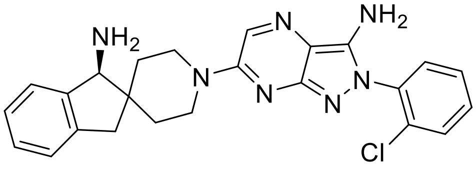

The title compound was synthesized following general procedure 2 using **15d** (101 mg, 0.23 mmol, 1.00 eq.) and 2-chlorophenylhydrazine hydrochloride (82 mg, 0.46 mmol, 2.00 eq.). The desired product was obtained as a yellow amorphous powder (26 mg, 20%). LC-MS (ESI) (m/z) $[M+H]^+$= 446.1; HRMS (ESI) (m/z): calcd for $C_{24}H_{24}ClN_7$ $[M+H]^+$ 446.1854, found 446.1889; ^1H NMR (300 MHz, MeOD) δ 8.41 (s, 1H), 7.70 (ddtd, *J* = 30.2, 15.0, 7.8, 1.6 Hz, 4H), 7.53 (d, *J* = 7.4 Hz, 1H), 7.48 – 7.32 (m, 3H), 4.68 – 4.41 (m, 3H), 3.52 (q, *J* = 13.3 Hz, 2H), 3.25 (s, 2H), 2.03 – 1.63 (m, 4H) ppm. ^{13}C NMR (75 MHz, MeOD) δ 155.59, 150.02, 147.22, 142.17, 137.21, 133.05, 132.79, 131.83, 131.45, 130.84, 130.48, 129.83, 128.57, 127.25, 125.65, 125.26, 110.43, 63.25, 44.66, 41.85, 41.63, 38.90, 34.34, 30.01 ppm.

(S)-1'-(3-Amino-2-(2,3-dichlorophenyl)-2H-pyrazolo[3,4-b]pyrazin-6-yl)-1,3-dihydrospiro[indene-2,4'-piperidin]-1-amine (17g)

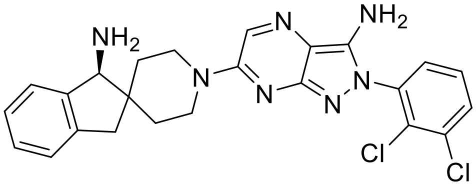

The tile compound was synthesized following general procedure 2 using **15d** (132 mg, 0.30 mmol, 1.00 eq.) and 2,3-dichlorophenylhydrazine hydrochloride (128 mg, 0.60 mmol, 2.00 eq.). The desired product was obtained as a yellow amorphous powder (41 mg, 23.6%); LC-MS (ESI) (m/z) $[M+H]^+$= 480.1; HRMS (ESI) (m/z): calcd for $C_{24}H_{23}Cl_2N_7$ $[M+H]^+$ 480.1465, found 480.1457; ^1H NMR (600 MHz, MeOD) δ 8.16 (d, *J* = 2.9 Hz, 1H), 7.78 (dt, *J* = 7.2, 2.9 Hz, 1H), 7.59 – 7.49 (m, 2H), 7.43 (dd, *J* = 7.1, 2.8 Hz, 1H), 7.33 – 7.22 (m, 3H), 4.51 – 4.37 (m, 2H), 4.13 (d, *J* = 2.9 Hz, 1H), 3.37 (d, *J* = 14.8 Hz, 2H), 3.20 (dd, *J* = 15.9, 2.8 Hz, 1H), 2.96 (dd, *J* = 15.9, 2.8 Hz, 1H), 1.87 (qd, *J* = 13.5, 3.8 Hz, 2H), 1.70 – 1.52 (m, 2H) ppm. ^{13}C NMR (151 MHz, MeOD) δ 168.85, 155.06, 151.36, 142.90, 142.15, 141.35, 137.02, 133.84, 131.70, 131.60, 129.59, 128.58, 128.18, 128.14, 126.62, 125.00, 124.31, 110.30, 64.17, 45.80, 42.35, 41.80, 39.06, 34.58, 28.95 ppm.

(S)-1'-(3-Amino-2-phenyl-2H-pyrazolo[3,4-b]pyrazin-6-yl)-1,3-dihydrospiro[indene-2,4'-piperidin]-1-amine (17h)

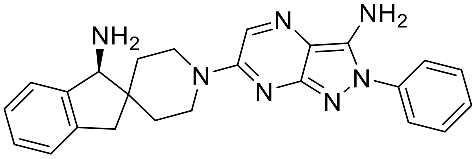

The title compound was synthesized following general procedure 2 using **15d** (44 mg, 0.1 mmol, 1.00 eq.) and phenylhydrazine-hydrochloride (29 mg, 0.20 mmol, 2.00 eq.). The desired product was obtained as a yellow amorphous powder (10 mg, 23%). LC-MS (ESI) (m/z) $[M+H]^+$= 412.1; HRMS (ESI) (m/z): calcd for $C_{24}H_{25}N_7$ $[M+H]^+$ 412.2244, found 412.2232; ^1H NMR (300 MHz, MeOD) δ 8.17 (s, 1H), 7.70 – 7.55 (m, 3H), 7.47 (td, *J* = 7.1, 5.4 Hz, 2H), 7.38 – 7.25 (m, 4H), 4.44 (dd, *J* = 25.0, 14.2 Hz, 2H), 4.25 (s, 1H), 3.41 (t, *J* = 11.3 Hz, 2H), 3.21 (d, *J* = 16.0 Hz, 1H), 3.06 (d, *J* = 16.6 Hz, 1H), 1.96 – 1.76 (m, 2H), 1.70 – 1.58 (m, 2H) ppm. ^{13}C NMR (75 MHz, MeOD) δ 155.00, 141.78, 138.28, 129.61, 129.34, 128.88, 127.89, 126.88, 125.30, 124.71, 124.21, 63.83, 47.32, 47.03, 46.75, 45.30, 42.17, 41.70, 38.95, 34.48, 29.39 ppm.

(S)-3-(3-Amino-6-(1-amino-1,3-dihydrospiro[indene-2,4'-piperidin]-1'-yl)-2H-pyrazolo[3,4-b]pyrazin-2-yl)benzonitrile (17i)

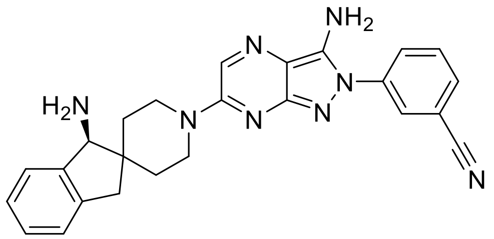

The title compound was synthesized following general procedure 2 using **15d** (44 mg, 0.10 mmol, 1.00 eq.) and 3-cyanophenylhydrazine hydrochloride (34 mg, 0.20 mmol, 2.00 eq.). The desired product was obtained as a yellow amorphous powder (15 mg, 34%). LC-MS (ESI) (m/z) $[M+H]^+$= 437.1; HRMS (ESI) (m/z): calcd for $C_{25}H_{24}N_8$ $[M+H]^+$ 437.2197, found 437.2201; ^1H NMR (300 MHz, MeOD) δ 8.37 (dd, *J* = 25.1, 1.4 Hz, 1H), 8.19 (s, 1H), 8.10 – 7.98 (m, 1H), 7.85 – 7.68 (m, 1H), 7.46 (t, *J* = 6.5 Hz, 1H), 7.36 – 7.27 (m, 4H), 4.55 – 4.34 (m, 2H), 4.22 (d, *J* = 17.3 Hz, 1H), 3.42 (dt, *J* = 11.3, 2.7 Hz, 2H), 3.21 (d, *J* = 15.8 Hz, 1H), 3.04 (t, *J* = 15.5 Hz, 1H), 1.90 – 1.76 (m, 2H), 1.65 (d, *J* = 13.2 Hz, 2H) ppm. ^{13}C NMR (75 MHz, MeOD) δ 155.02, 154.05, 151.37, 147.23, 141.90, 141.60, 139.39, 131.63, 130.93, 130.55, 130.24, 128.91, 128.30, 127.18, 126.94, 125.29, 124.72, 113.33, 63.67, 50.46, 38.94, 34.48, 29.58, 29.26, 27.45 ppm.

(S)-4-(3-Amino-6-(1-amino-1,3-dihydrospiro[indene-2,4'-piperidin]-1'-yl)-2H-pyrazolo[3,4-b]pyrazin-2-yl)benzonitrile (17j)

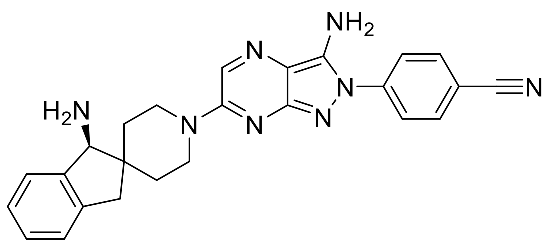

The title compound was synthesized following general procedure 2 using **15d** (44 mg, 0.1 mmol, 1.00 eq.) and 4-cyanophenylhydrazine hydrochloride (34 mg, 0.20 mmol, 2.00 eq.). The desired product was obtained as a yellow amorphous powder (12 mg, 27%). LC-MS (ESI) (m/z) $[M+H]^+$= 437.1; HRMS (ESI) (m/z): calcd for $C_{25}H_{24}N_8$ $[M+H]^+$ 437.2197, found 437.2182; ^1H NMR (300 MHz, MeOD) δ 8.54 (s, 1H), 8.38 (dd, *J* = 25.0, 1.4 Hz, 1H), 8.20 (s, 1H), 7.92 (s, 2H), 7.50 (d, *J* = 7.2 Hz, 1H), 7.37 (d, *J* = 10.4 Hz, 3H), 4.60 – 4.24 (m, 3H), 3.50 – 3.38 (m, 2H), 3.27 – 3.01 (m, 2H), 1.99 – 1.80 (m, 2H), 1.69 (t, *J* = 15.8 Hz, 2H) ppm. ^{13}C NMR (151 MHz, MeOD) δ 155.05, 151.57, 147.20, 142.39, 142.08, 141.84, 133.84, 133.35, 130.42, 129.47, 127.09, 125.52, 125.03, 123.91, 117.80, 111.68, 110.47, 63.54, 63.49, 44.92, 42.03, 38.93, 38.82, 34.60, 34.45 ppm.

Ethyl (S)-3-(3-Amino-6-(1-amino-1,3-dihydrospiro[indene-2,4'-piperidin]-1'-yl)-2H-pyrazolo[3,4-b]pyrazin-2-yl)benzoate (17k)

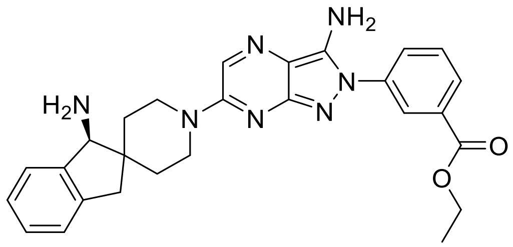

The title compound was synthesized following general procedure 2 using **15d** (66 mg, 0.15 mmol, 1.00 eq.) and ethyl 3-hydrazinobenzoate hydrochloride (65 mg, 0.30 mmol, 2.00 eq.). The desired product was obtained as a yellow amorphous powder (13 mg, 18%). LC-MS (ESI) (m/z) $[M+H]^+$= 484.1; HRMS (ESI) (m/z): calcd for $C_{27}H_{29}N_7O_2$ $[M+H]^+$ 484.2455, found 484.2475; ^1H NMR (300 MHz, MeOD) δ 8.32 (t, *J* = 1.9 Hz, 1H), 8.19 (s, 1H), 8.11 (dt, *J* = 7.9, 1.3 Hz, 1H), 8.02 – 7.90 (m, 1H), 7.71 (t, *J* = 7.9 Hz, 1H), 7.52 (d, *J* = 7.2 Hz, 1H), 7.46 – 7.34 (m, 3H), 7.25 (s, 1H), 4.60 – 4.34 (m, 4H), 3.44 (dd, *J* = 19.0, 11.4 Hz, 1H), 3.22 (d, *J* = 2.6 Hz, 1H), 3.17 – 3.04 (m, 1H), 1.99 – 1.62 (m, 4H), 1.56 – 1.39 (m, 5H) ppm. ^{13}C NMR (151 MHz, MeOD) δ 167.93, 165.60, 155.02, 151.09, 142.22, 138.73, 137.60, 129.89, 129.67, 129.62, 128.42, 128.37, 127.91, 127.15, 125.61, 125.11, 124.96, 124.76, 111.54, 63.51, 61.20, 42.04, 38.91, 27.37, 27.26, 26.79, 26.18, 13.16 ppm.

(S)-3-(3-Amino-6-(1-amino-1,3-dihydrospiro[indene-2,4'-piperidin]-1'-yl)-2H-pyrazolo[3,4-b]pyrazin-2-yl)benzoic acid (17l)

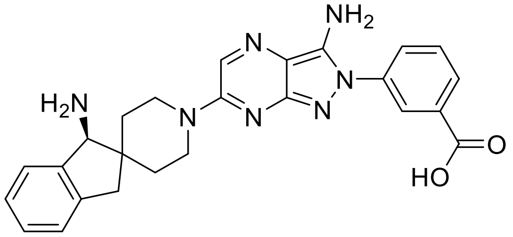

To a solution of **17k** (10mg, 0.02 mmol, 1.00 eq.) in MeOH (2 mL) was added 0.5 mL of a 2M NaOH solution. The mixture was stirred at room temperature for 4h. After evaporating the solvent under reduced pressure, the obtained crude product was then purified by reversed-phase preparative HPLC (C18 column, solvent A: H_2O/0.1% TFA, solvent B: ACN/0.1% TFA. Gradient 5-45% solvent B over 30 min) to achieve after lyophilisation the desired product as a yellow amorphous powder (3mg, 35%). LC-MS (ESI) (m/z) $[M+H]^+$= 458.2; HRMS (ESI) (m/z): calcd for $C_{25}H_{25}N_7O_2$ $[M+H]^+$ 458.2299, found 458.2257; ^{1}H NMR (600 MHz, DMSO-d_6) δ 8.31 (s, 1H), 8.18 (d, *J* = 9.0 Hz, 1H), 7.88 (dt, *J* = 7.6, 1.4 Hz, 1H), 7.77 (d, *J* = 7.9 Hz, 1H), 7.55 (t, *J* = 7.8 Hz, 1H), 7.38 – 7.31 (m, 1H), 7.26 – 7.17 (m, 2H), 6.44 (s, 1H), 4.41 – 4.28 (m, 2H), 3.93 (s, 1H), 3.25 – 3.18 (m, 3H), 3.12 (d, *J* = 15.7 Hz, 1H), 2.71 (d, *J* = 15.7 Hz, 1H), 1.82 (td, *J* = 12.7, 4.2 Hz, 1H), 1.72 (td, *J* = 12.6, 4.2 Hz, 1H), 1.56 – 1.51 (m, 1H), 1.24 (s, 1H), 1.19 (d, *J* = 13.4 Hz, 1H) ppm. ^{13}C NMR (151 MHz, DMSO-d_6) δ 167.57, 154.70, 150.63, 141.51, 141.27, 139.34, 129.92, 129.33, 127.88, 127.58, 126.75, 125.17, 124.62, 124.53, 112.64, 64.83, 47.06, 43.03, 42.13, 40.53, 34.94, 28.38 ppm.

(S)-1'-(2-(2-Chlorophenyl)-2H-pyrazolo[3,4-b]pyrazin-6-yl)-1,3-dihydrospiro[indene-2,4'-piperidin]-1-amine (18)

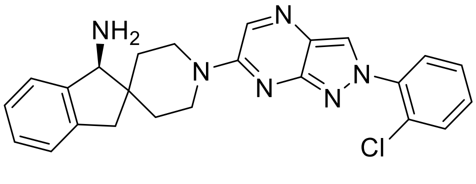

To a solution of Boc-protected **17f** (11 mg, 0.020 mmol, 1.00 eq.) in THF (2mL) was added *t*BuONO (5.5 mg, 0.054 mmol, 2.70 eq.). The mixture was stirred at 66°C for 2 h. After cooling to room temperature, the reaction mixture was poured into a separatory funnel containing water (10 mL) and was extracted three times with DCM (5 x 3 mL). The combined organic phase was dried over $MgSO_4$ and concentrated under reduced pressure. The obtained Boc-protected crude

intermediate was deprotected by stirring in 20% TFA in DCM (v/v, 1 mL) for 2h. The solution was co-evaporated with toluene twice to give the crude primary amine TFA salt. The crude product was then purified by reversed-phase preparative HPLC (C18 column, solvent A: H_2O/0.1% TFA, solvent B: ACN/0.1% TFA. Gradient 5-55% solvent B over 35 min) to achieve, after lyophilisation, the desired product as a yellow amorphous powder (3mg, 33%). LC-MS (ESI) (m/z) $[M+H]^+$= 431.2; HRMS (ESI) (m/z): calcd for $C_{24}H_{23}ClN_6$ $[M+H]^+$ 431.1745, found 431.1831; ^1H NMR (600 MHz, MeOD) δ 8.60 (d, *J* = 10.5 Hz, 2H), 7.70 (tq, *J* = 6.4, 3.1 Hz, 2H), 7.61 – 7.54 (m, 2H), 7.52 (d, *J* = 7.6 Hz, 1H), 7.47 – 7.41 (m, 2H), 7.37 (td, *J* = 7.1, 2.3 Hz, 1H), 4.62 – 4.57 (m, 1H), 4.49 – 4.45 (m, 1H), 4.43 (s, 1H), 3.53 (ddd, *J* = 14.2, 11.5, 2.9 Hz, 1H), 3.49 – 3.43 (m, 1H), 3.25 (s, 2H), 1.99 – 1.91 (m, 1H), 1.87 (ddd, *J* = 15.5, 11.7, 4.3 Hz, 1H), 1.83 – 1.77 (m, 1H), 1.70 (dq, *J* = 13.7, 2.9 Hz, 1H) ppm. ^{13}C NMR (151 MHz, MeOD) δ 154.10, 151.22, 142.25, 138.07, 137.25, 136.66, 130.50, 130.47, 129.81, 129.17, 128.23, 127.83, 127.22, 127.07, 125.67, 125.15, 124.04, 63.45, 48.16, 44.73, 42.18, 41.73, 38.92, 34.39, 29.88 ppm.

***tert*-Butyl (S)-(1'-(4-chloro-5-cyanopyrimidin-2-yl)-1,3-dihydrospiro[indene-2,4'-piperidin]-1-yl)carbamate (20a)**

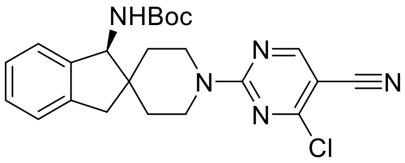

The title compound was synthesized following general procedure 1 using 2,4-dichloropyrimidine-5-carbonitrile (174 mg, 1.00 mmol, 1.00 eq.) and (1S)-1,3-dihydrospiro[indene-2,4'-piperidin]-1-amine dihydrochloride (413 mg, 1.50 mmol, 1.50 eq.). The desired product was obtained as a yellow oil (242 mg, 55%). LC-MS (ESI) (m/z) $[M+H]^+$= 440.1; HRMS (ESI) (m/z): calcd for $C_{23}H_{26}ClN_5O_2$ $[M+H]^+$ 440.1848, found 440.1821; ^1H NMR (300 MHz, MeOD) δ 8.56 (s, 1H), 7.22 (d, J = 6.8 Hz, 4H), 4.63 (s, 1H), 4.46 (s, 1H), 3.66 (d, J = 2.1 Hz, 1H), 3.54 (s, 2H), 3.15 (d, J = 16.3 Hz, 1H), 2.84 (d, J = 15.9 Hz, 1H), 1.73 (t, J = 14.7 Hz, 3H), 1.50 (d, J = 6.8 Hz, 9H) ppm. ^{13}C NMR (75 MHz, $CDCl_3$) δ 155.85, 153.05, 150.38, 141.54, 140.39, 128.54, 128.50, 127.35, 125.26, 124.76, 115.80, 113.60, 80.07, 62.91, 46.17, 42.40, 41.86, 40.59, 35.22, 29.79, 28.38 ppm.

***tert*-Butyl (S)-(1'-(6-chloro-5-cyanopyridin-2-yl)-1,3-dihydrospiro[indene-2,4'-piperidin]-1-yl)carbamate (20b)**

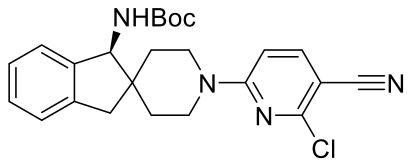

The title compound was synthesized following general procedure 1 using 2,6-dichloronicotinonitrile (100 mg, 0.60 mmol, 1.00 eq.) and (1S)-1,3-dihydrospiro[indene-2,4'-piperidin]-1-amine dihydrochloride (159 mg, 0.90 mmol, 1.50 eq.). The desired product was obtained as a colorless oil (105 mg, 41%). LC-MS (ESI) (m/z) $[M+H]^+$= 439.1; HRMS (ESI) (m/z): calcd for $C_{23}H_{27}ClN_4O_2$ $[M+H]^+$ 439.1895, found 439.1894; ^1H NMR (300 MHz, MeOD) δ 7.22 (d, J = 6.0 Hz, 6H), 3.85 (tt, J = 14.4, 4.1 Hz, 2H), 3.14 (d, J = 14.6 Hz, 2H), 3.07 (d, J = 15.8 Hz, 2H), 2.75 (d, J = 15.8 Hz, 1H), 1.81 – 1.56 (m, 4H), 1.51 (s, 9H) ppm. ^{13}C NMR (75 MHz, MeOD) δ 157.22, 155.18, 142.16, 141.95, 140.87, 127.61, 127.52, 126.42, 124.69, 124.01, 123.91, 104.37, 79.57, 78.96, 78.89, 63.05, 47.32, 47.04, 46.75, 39.94, 39.54, 35.49, 35.15, 29.43, 27.40, 27.36, 27.31 ppm.

***tert*-Butyl (S)-(1'-(3-chloro-4-cyanophenyl)-1,3-dihydrospiro[indene-2,4'-piperidin]-1-yl)carbamate (20c)**

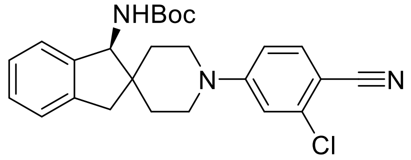

To a solution of 2-chloro-4-fluorobenzonitrile (70 mg, 0.45 mmol, 1.00 eq.) and (1S)-1,3-dihydrospiro[indene-2,4'-piperidin]-1-amine dihydrochloride (124 mg, 0.45 mmol, 1.00 eq.) in DMF (3mL) was added DIPEA (314 µL, 1.80 mmol, 4.00 eq.). The reaction mixture was stirred at 85°C for 6h. Then Boc_2O (196 mg, 0.90 mmol, 2.00 eq.) was added at room temperature and let stir for another 2h. After completion, the mixture was then diluted with DCM (15 mL) and washed with water (10 mL) and brine (10 mL). The organic phase was dried over $MgSO_4$, filtered, and concentrated under reduced pressure. The obtained crude product was purified by column chromatography (SiO_2, 0% to 100% ethyl acetate in cyclohexane) to afford the desired product as a colorless oil after evaporation of the pure fractions (150mg, 79%). LC-MS (ESI) (m/z) $[M+H]^+$= 438.1; HRMS (ESI) (m/z): calcd for $C_{25}H_{28}ClN_3O_2$ $[M+H]^+$ 438.1943, found 438.1930; ^1H NMR (300 MHz, MeOD) δ 7.51 (d, J = 8.9 Hz, 1H), 7.22 (s, 4H), 7.06 (d, J = 2.5 Hz, 1H), 6.94 (dd, J = 9.0, 2.5 Hz, 1H), 3.77 (ddt, J = 14.1, 9.1, 3.4 Hz, 2H), 3.24 (dddd, J = 13.6, 10.8, 5.3, 3.3 Hz, 2H), 3.11 (d, J = 15.7 Hz, 1H), 2.79 (d, J = 15.8 Hz, 1H), 1.95 – 1.61 (m, 4H), 1.47 (s, 9H) ppm. ^{13}C

NMR (75 MHz, MeOD) δ 157.22, 154.09, 141.93, 140.85, 137.46, 134.46, 127.58, 126.48, 124.74, 123.94, 117.01, 113.74, 112.19, 98.25, 78.94, 63.03, 46.21, 44.49, 43.99, 39.78, 34.76, 28.86, 27.38 ppm.

(S)-1'-(3-Amino-2-(2-chlorophenyl)-2H-pyrazolo[3,4-d]pyrimidin-6-yl)-1,3-dihydrospiro[indene-2,4'-piperidin]-1-amine (21a)

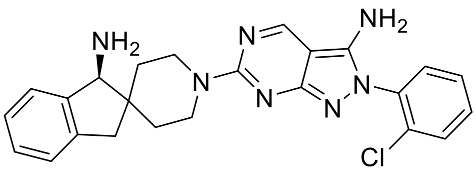

The title compound was synthesized following general procedure 2 using **20a** (110 mg, 0.25 mmol, 1.00 eq.) and 2-chlorophenylhydrazine hydrochloride (90 mg, 0.50 mmol, 2.00 eq.). The desired product was obtained as a yellow amorphous powder (7 mg, 5%). LC-MS (ESI) (m/z) $[M+H]^+$= 446.1; HRMS (ESI) (m/z): calcd for $C_{24}H_{24}ClN_7$ $[M+H]^+$ 446.1854, found 446.1863; ^1H NMR (300 MHz, MeOD) δ 8.41 (s, 1H), 7.70 (ddtd, *J* = 30.2, 15.0, 7.8, 1.6 Hz, 4H), 7.53 (d, *J* = 7.4 Hz, 1H), 7.48 – 7.32 (m, 3H), 4.68 – 4.41 (m, 3H), 3.52 (q, *J* = 13.3 Hz, 2H), 3.25 (s, 2H), 2.03 – 1.63 (m, 4H) ppm. ^{13}C NMR (75 MHz, MeOD) δ 159.48, 156.28, 151.32, 150.54, 142.25, 137.26, 135.80, 132.89, 131.24, 129.72, 128.80, 128.58, 127.14, 125.61, 125.14, 99.89, 63.39, 44.69, 41.04, 40.94, 38.73, 34.26, 29.96 ppm.

(S)-1'-(3-Amino-2-(2,3-dichlorophenyl)-2H-pyrazolo[3,4-d]pyrimidin-6-yl)-1,3-dihydrospiro[indene-2,4'-piperidin]-1-amine (21b)

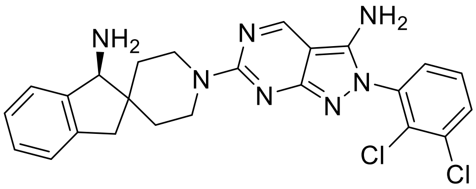

The title compound was synthesized following general procedure 2 using **20a** (84 mg, 0.20 mmol, 1.00 eq.) and 2,3-dichlorophenylhydrazine hydrochloride (82 mg, 0.40 mmol, 2.00 eq.). The desired product was obtained as a yellow amorphous powder (10 mg, 10%); LC-MS (ESI) (m/z) $[M+H]^+$= 480.1; HRMS (ESI) (m/z): calcd for $C_{24}H_{23}Cl_2N_7$ $[M+H]^+$ 480.1465, found 480.1503; ^1H NMR (600 MHz, MeOD) δ 8.83 (s, 1H), 7.60 – 7.57 (m, 1H), 7.50 (ddd, J = 14.2, 8.2, 6.3 Hz, 2H), 7.44 – 7.30 (m, 4H), 4.65 (d, J = 13.6 Hz, 1H), 4.55 (d, J = 13.9 Hz, 1H), 4.37 (s, 1H), 3.36 (d, J = 3.0 Hz, 1H), 3.31 – 3.24 (m, 2H), 3.17 (d, J = 3.2 Hz, 2H), 1.81 – 1.65 (m, 4H) ppm. ^{13}C NMR (151 MHz, MeOD) δ 155.45, 150.53, 142.17, 137.18, 135.76, 135.74, 133.27, 131.16, 129.83,

129.29, 127.24, 125.65, 125.21, 110.11, 63.31, 44.69, 41.97, 41.68, 38.93, 34.36, 29.97, 25.35, 24.37 ppm.

(S)-1'-(3-Amino-2-(2,3-dichlorophenyl)-2H-pyrazolo[3,4-b]pyridin-6-yl)-1,3-dihydrospiro[indene-2,4'-piperidin]-1-amine (21c)

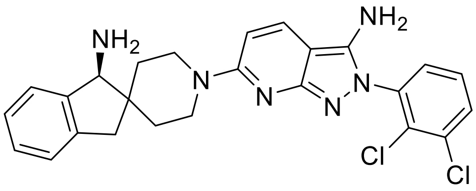

The title compound was synthesized following general procedure 2 using **20b** (100 mg, 0.23 mmol, 1.00 eq.) and 2,3-dichlorophenylhydrazine hydrochloride (80 mg, 0.46 mmol, 2.00 eq.)**.** The desired product was obtained as a yellow amorphous powder (10 mg, 9%). LC-MS (ESI) (m/z) $[M+H]^+$= 479.1; HRMS (ESI) (m/z): calcd for $C_{25}H_{24}Cl_2N_6$ $[M+H]^+$ 479.1512, found 479.1525; ^{1}H NMR (300 MHz, MeOD) δ 7.99 – 7.69 (m, 2H), 7.61 – 7.24 (m, 5H), 6.63 (d, J = 9.2 Hz, 1H), 4.36 (q, J = 19.5 Hz, 3H), 3.75 – 3.54 (m, 2H), 3.19 (s, 2H), 2.00 – 1.52 (m, 4H) ppm. ^{13}C NMR (75 MHz, MeOD) δ 160.30, 158.22, 143.38, 142.37, 137.57, 137.13, 133.78, 131.70, 131.53, 129.66, 128.67, 128.12, 127.11, 125.61, 125.15, 102.59, 94.39, 63.52, 44.86, 42.38, 41.95, 38.95, 34.51, 29.98 ppm.

((S)-1'-(3-Amino-2-(2,3-dichlorophenyl)-2H-indazol-6-yl)-1,3-dihydrospiro[indene-2,4'-piperidin]-1-amine (21d)

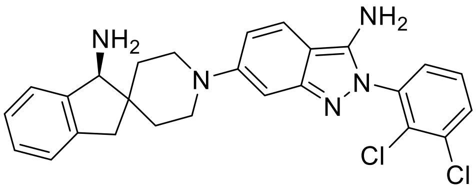

The title compound was synthesized following general procedure 2 using **20c** (100 mg, 0.23 mmol, 1.00 eq.) and 2,3-dichlorophenylhydrazine hydrochloride (80 mg, 0.46 mmol, 2.00 eq.)**.** The desired product was obtained as a yellow amorphous powder (20 mg, 18%). LC-MS (ESI) (m/z) $[M+H]^+$ = 478.1; HRMS (ESI) (m/z): calcd for $C_{26}H_{24}Cl_2N_5$ $[M+H]^+$ 478.1560, found 478.1541; ^{1}H NMR (300 MHz, MeOD) δ 7.78 (p, J = 3.9 Hz, 1H), 7.59 – 7.46 (m, 4H), 7.40 – 7.26 (m, 3H), 6.75 (dd, J = 9.2, 1.9 Hz, 1H), 6.65 (d, J = 1.8 Hz, 1H), 4.34 (s, 1H), 3.71 – 3.53 (m, 2H), 3.21 – 2.97 (m, 4H), 2.08 – 1.83 (m, 2H), 1.70 (dd, J = 25.8, 13.0 Hz, 2H) ppm. ^{13}C NMR (75 MHz, MeOD) δ 168.87, 152.44, 150.57, 143.18, 142.23, 138.57, 137.38, 133.77, 131.62, 129.34,

128.57, 128.10, 126.98, 125.50, 125.04, 120.95, 113.40, 103.28, 96.99, 63.65, 44.40, 38.97, 34.66, 30.04 ppm.

Biochemical SHP2 assay

A fluorescence-based assay to measure inhibition against SHP2 was performed in 384-well plates. Briefly, the tested compounds were solubilized in DMSO and serially diluted into the indicated concentrations for the biological test. Enzymatic activities of SHP2 were determined at room temperature by monitoring the dephosphorylation of substrate 6,8-Difluoro-4-Methylumbelliferyl Phosphate (DiFMUP); products were then detected at a 355 nm excitation wavelength and 460 nm emission wavelength by the EnVision multilabe plate reader (PerkinElmer). The assays were carried out in a final volume of 50 µL containing 60 mM HEPES, pH 7.2, 75 mM NaCl, 75 mM KCl, 1 mM EDTA, 0.05% Tween-20, 5 mM DTT, 2 nM enzyme, and the tested compound at indicated concentrations. SHP2 was co-incubated with 5 µM of bisphosphorylated IRS1 peptide (sequence: H2N-LN(pY)IDLDLV(dPEG8)LST(pY)ASINFQK-amide) and tested compounds. After 30 min incubation at room temperature, the substrate (DiFMUP) was added to a final concentration of 25 µM. The initial rate of the dephosphorylation was presented by the early linear region of the enzymatic reaction kinetic curve, and the inhibitory activity of the compound was continuously monitored. The inhibitor dose−response curves were analyzed using GraphPad Prism software version 8 (GraphPad Software Inc) with a variable slope (four parameters).

Growth inhibition MV-4-11 cells

MV-4-11 or MV-4-11 SHP2^{E76K} (10000 cells/well) were plated onto 96-well plates (PerkinElmer Culture Plate-96, White Opaque 96-well Microplate) in 100 µL of medium (IMDM containing 10% fetal bovine serum, Gibco). Compounds at the indicated concentrations were added 2 h after cell plating. At day 3, 10 µL of CCK8 (MeilunBio) was added, and the signal was determined according to the supplier’s protocol by using SpectraMax 190 Microplate Reader.

Construction of SHP2 mutated cells

For MV-4-11 cells harboring the SHP2^{E76K} mutation, cDNA encoding eGFP-fused SHP2^{E76K} mutation was generated by PCR and cloned into a lentiviral expression vector (pLenti CMV Puro DEST) that carried a puromycin-resistant gene. In detail, MV-4-11 cells were cultured in IMDM/10% fetal bovine serum (FBS) and infected with lentiviruses encoding eGFP-fusion SHP2^{E76K}. Infected cells were selected with puromycin (1 µg/ml). Expression of eGFP fusion SHP2^{E76K} in MV-4-11 cells was verified by immunoblotting with SHP2 antibody.

pERK cellular assay MV-4-11 cells

The cellular p-ERK inhibition activity was measured by AlphaScreen SureFire Phospho-ERK 1/2 Kit (PerkinElmer). MV-4-11 cells (30,000 cells/well) were seeded in a 96-well plate and incubated for 30 min, then treated with SHP2 inhibitors at concentrations of 1, 0.33, 0.11, 0.037, 0.012, 0.004, and 0.001 μM for 1h at 37°C. Incubations were terminated by the addition of 12.5 μL of lysis buffer supplied with the SureFire phospho-extracellular signal-regulated kinase (p-ERK) assay kit. Samples were processed according to the manufacturer's directions. The fluorescence signal from p-ERK was measured in duplicate using EnVision multilane plate reader (PerkinElmer). The percentage of inhibition was normalized by the total pERK signal and compared with the DMSO vehicle control.

pERK cellular assay Panc-1 and KYSE520 cells

The measurement of cellular pERK levels was performed via in-cell western technology using a Licor Odyssey M scanner. Cells (Panc-1 or KYSE520) were seeded in clear, flat-bottomed 96-well plates and left to attach overnight in the incubator (37 °C, 5% CO_2, 95% humidity). The cells were then treated with vehicle control (DMSO), inhibitor control (100 nM trametinib), reference compound (SHP099 or TNO155), or the novel SHP2i compounds described above. Treated cells were left to incubate at 37 °C for 1 h. After 1 hour, the cells were taken from the incubator, the treatment was removed, and the in-cell western protocol started. Cells were fixed in 4% PFA for 20 minutes at room temperature. After removal of the fixation buffer, cells were permeabilized in TBS containing 0.1% Triton X for 20 minutes (RT, gentle shaking). Cells were then incubated with primary antibodies against ERK 1/2 and pERK (sc-514302 (1:250) and cst4370 (1:500), respectively) overnight at 4 °C with gentle shaking. The next day, the antibody solution was removed, and the wells were washed 4x with TBST for 5 minutes each. After those cells were incubated for 1 hour with a secondary antibody mixture containing IRDye® 800CW Goat anti-Rabbit (1:800), IRDye® 680RD Goat anti-Mouse (1:800), and CellTag™ 520 Stain (1:800) in Antibody Diluent (Licor). The wells were then washed again (4x, 5 min). Plates were scanned on the Licor Odyssey M scanner using the Licor Aquistion software. Analysis was performed using Empiria studio software and GraphPad Prism.

SHP2 overexpression, purification

Human SHP2 (amino acids 2-525, $SHP2_{(2-525)}$) with a TEV-cleavable N-terminal His_7-tag was expressed in *E.coli* Rosetta DE3 from the pQlinkH vector. Cells were grown in TB-medium at 37 °C to an OD_{600nm} of 0.8, the temperature was lowered to 18 °C, and protein expression was

induced by the addition of 500 µM isopropyl β-D-1-thiogalactopyranoside (IPTG). Following overnight expression, cells were harvested by 15 min centrifugation at 7,000 rpm, resuspended in PBS, 0.5 M NaCl, 5% glycerol, 1 µg/ml lysozyme, 0.1% Triton X-100, 2 µg/ml DNAseI, 250 µM Pefabloc, and lysed by two freeze and thaw cycles. The lysate was centrifuged at 22,500 rpm for 45 min at 10 °C. The supernatant was supplemented with 15 mM imidazole and applied to a HisTrap™ High Performance Ni-immobilized metal chelate affinity chromatography (Ni-IMAC) column (GE Healthcare) equilibrated with buffer A (20 mM Tris pH 8, 0.5 M NaCl). Bound SHP2 was eluted by an imidazole gradient from 0 to 250 mM. Fractions with the target protein were pooled, His-TEV-protease was added, and the protein was dialyzed overnight at 4 °C against buffer A. TEV-cleaved SHP2 was re-applied to the Ni-IMAC column, the non-bound SHP2 fractions were collected and concentrated for application to the size exclusion column HiLoad™ Superdex S200 16/600 (GE Healthcare) in 20 mM Tris pH 8, 150 mM NaCl, 2 mM DTT running buffer. SHP2 fractions were pooled and concentrated to 15 mg/ml. Purification success was checked by SDS PAGE.

Crystallization and structure determination

$SHP2_{(2\text{-}525)}$ was combined with 1 mM compound **17g** containing 4% DMSO in total and crystallized employing the sitting-drop vapor-diffusion method by mixing 200 nl of complex solution and an equal volume of crystallization solution containing 20% w/v PEG3350, 0.2 M sodium acetate, 0.1 M Bis-Tris propane pH 8.5. Experiments were assisted by a Gryphon pipetting robot (Matrix Technologies Co.) and Rock imager 1000 storage system (Formulatrix). Crystals appeared within 7 days and were transferred into a cryo-protection solution containing the crystallization solution supplemented with 20% ethylene glycol before flash freezing in liquid nitrogen. Diffraction data were recorded to 1.87 Å at BL14.1 at BESSY II (Helmholtz-Zentrum Berlin, HZB), processed and scaled using XDSapp.[2] The complex structure was solved by molecular replacement with Phaser using the SHP2 structure pdb 5EHR as a search model.[3] The structure was manually adjusted using COOT[4] and iteratively refined using Phenix[5] to an R_{work} of 18.4% and an R_{free} of 22.9% (**Supplementary Table S1**).

Data availability

The atomic coordinates of $SHP2_{(1\text{-}525)}$ with bound compound **17g** have been deposited in the Protein Data Bank under accession code 9TKU. All other data relevant to this work are available from the authors upon request.

Molecular docking studies

Molecular docking was performed using Schrodinger Maestro 14.2. Protein structure with PDB ID 6WU8 was prepared with Maestro's Protein Preparation Workflow, at a pH of 7.4, using default settings. Ligand structures were prepared using LigPrep and the ionization states expected at pH 7.4. Receptor grids were set by using the ligand in the appropriate protein structure as a reference, which included the tunnel site. The target ligand was subsequently docked into the SHP2 allosteric site using Glide's extra-precision (XP) mode. The docking images were generated by PyMOL.

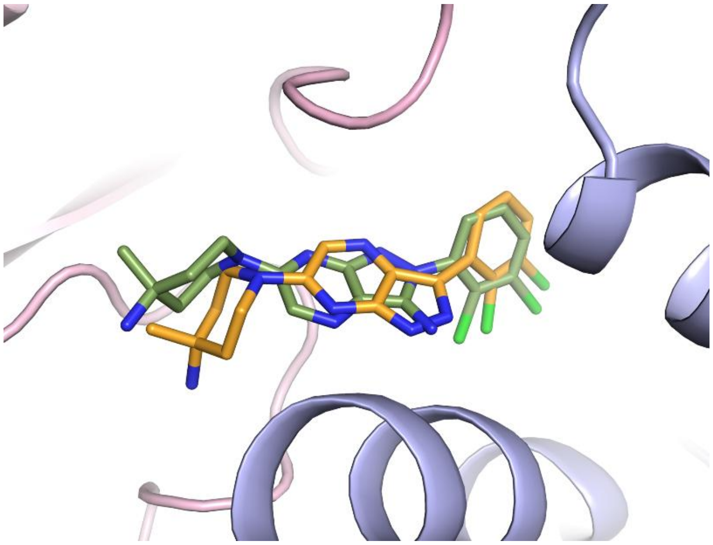

Supplementary Figure S1: 3D overlay of IACS-13909 **6** (orange) and compound **13** (green) in the SHP2 allosteric binding site (PDB ID: 6WU8).

Isothermal titration calorimetry (ITC)

ITC experiments were performed using a PEAQ-ITC titration microcalorimeter (GE Healthcare, Freiburg, Germany) at 18 °C. 500 μM compound **17g** was titrated to 20 μM $SHP2_{(2-525)}$, both dialyzed before against 20 mM Tris pH 7.5, 150 mM NaCl, 2 mM DTT, 10% DMSO. Raw data (incremental heat per molecule of added ligand) were fitted by nonlinear least squares with the ORIGIN7 software using a one-site binding model.

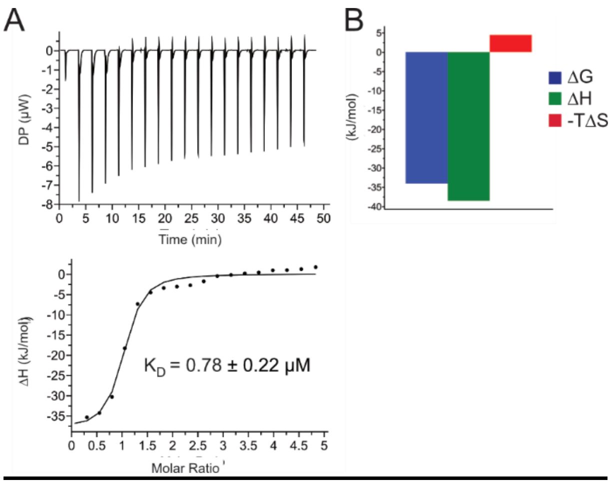

Supplementary Figure S2: A) Isothermal titration calorimetry of compound **17g** titrated to full-length SHP2. The data were fitted to a one-site binding model showing a binding affinity of 0.95 ± 0.22 μM with a binding number n = 0.95 ± 0.03. B) The signature plot shows a ΔG = -34.1 kJ/mol; ΔH = -38.5 ± 1.7 kJ/mol and -TΔS = 4.5 kJ/mol of the ITC titration shown in A).

Measurement of the kinetic solubility

The UV absorbance of the corresponding compound was measured by LC-MS using UV detection at a wavelength of 254 nm. Calibration curves were generated by preparing serial dilutions (200 μM to 12.5 μM) of each compound in acetonitrile, and regression equations were obtained by plotting the UV absorbance area against concentration. Then, a saturated solution of each compound was prepared in PBS buffer (pH = 7.4). The samples were sonicated for 5 min and incubated at room temperature on a shaker for 24 hours. After incubation, the samples were centrifuged and filtered, and the UV absorbance of the supernatant was measured by LC-MS. The kinetic solubility of each compound was calculated from the corresponding calibration curve regression equation.

NMR spectra (^{1}H-NMR and ^{13}C-NMR) spectra of key compounds and intermediates

Compound **15a (^{1}H-NMR, CDCl$_3$, 300 MHz)**

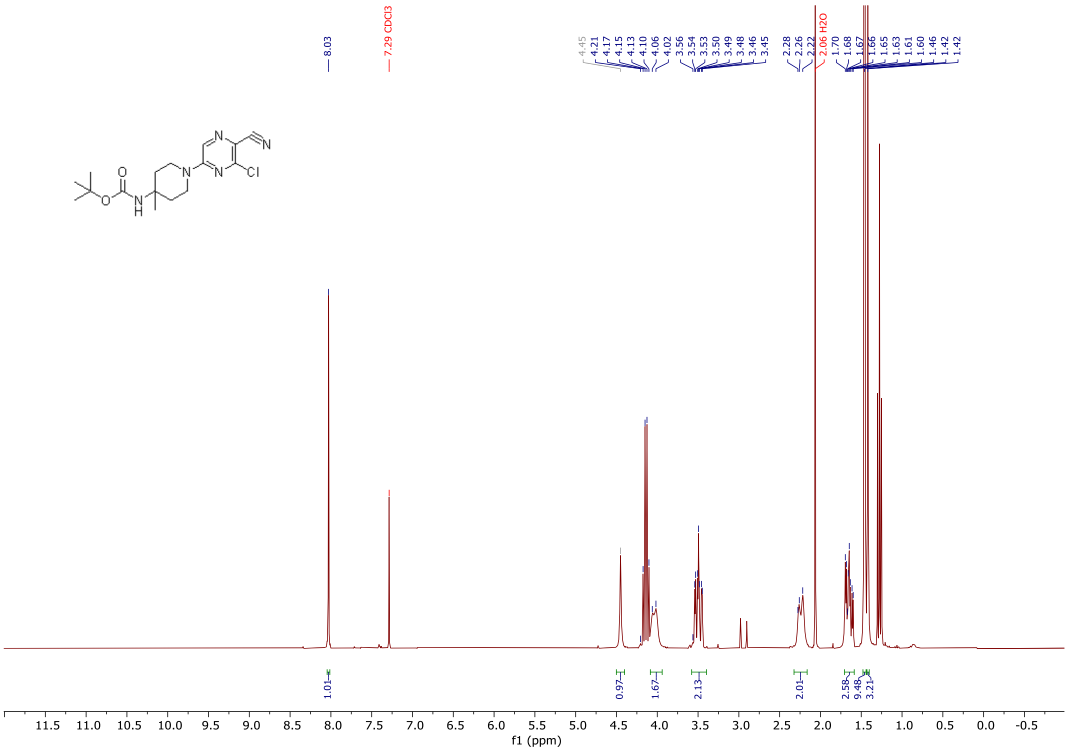

Compound **15a (^{13}C-NMR, CDCl$_3$, 75 MHz)**

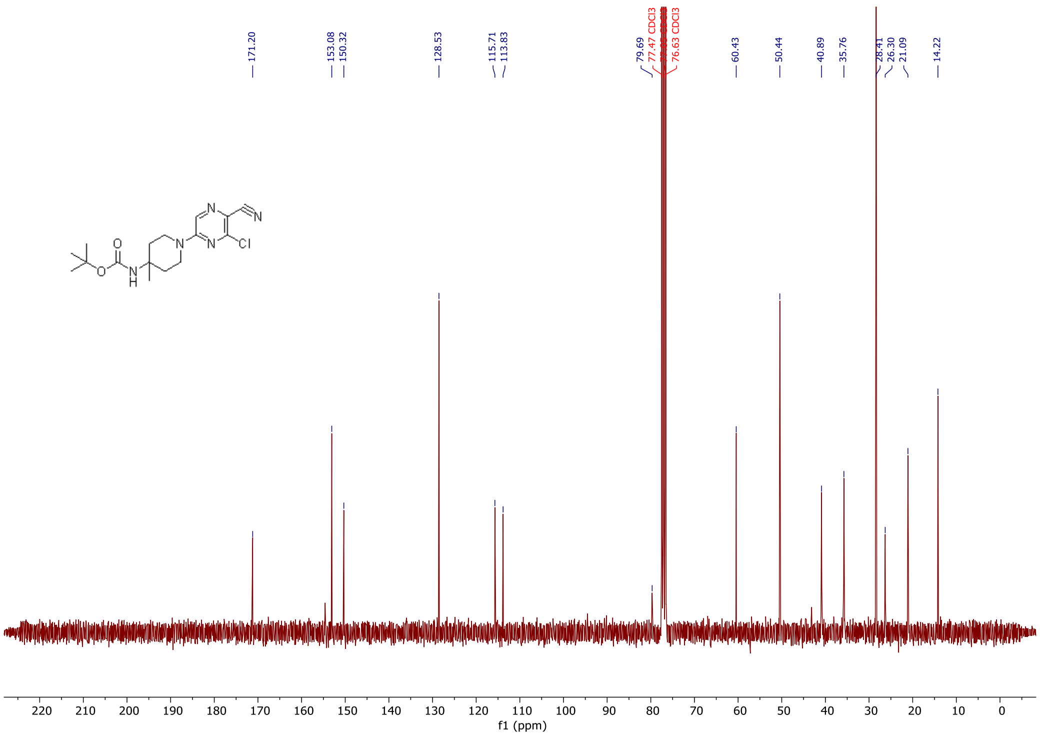

Compound **15d** (^{1}H-NMR, $CDCl_3$, 300 MHz)

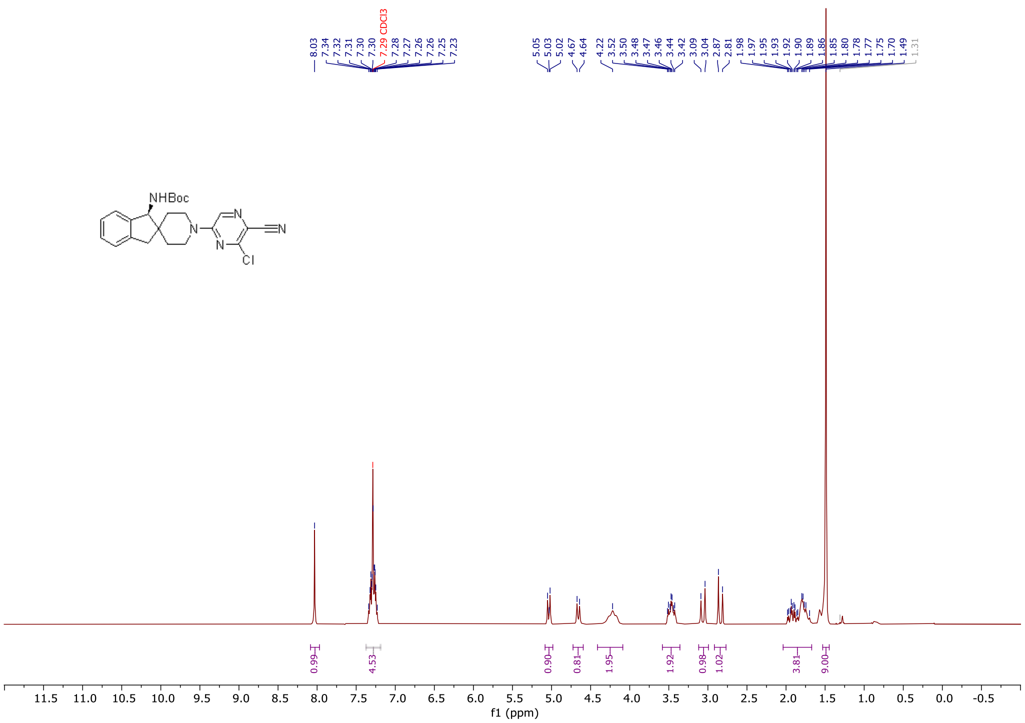

Compound **15d** (^{13}C-NMR, $CDCl_3$, 75 MHz)

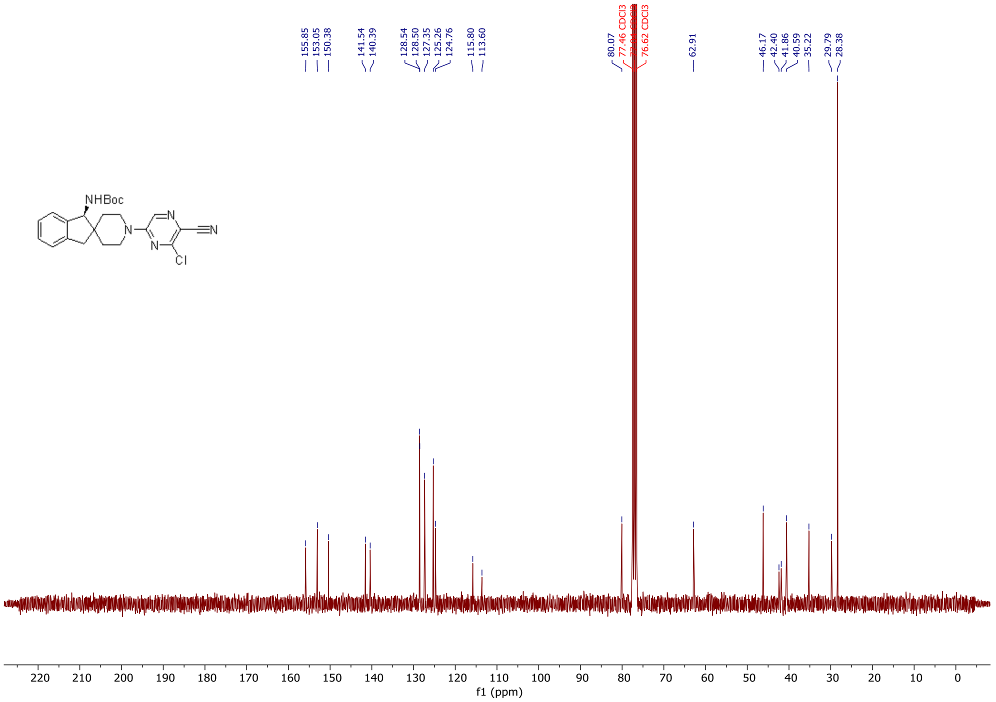

Compound **15e (¹H-NMR, MeOD, 300 MHz)**

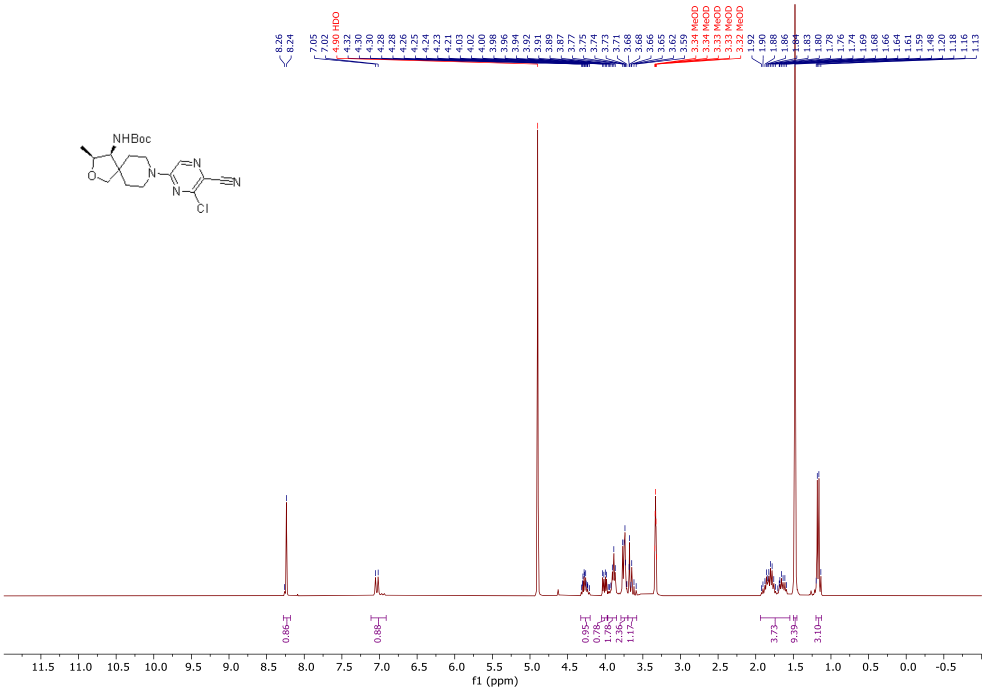

Compound **15e (¹³C-NMR, MeOD, 75 MHz)**

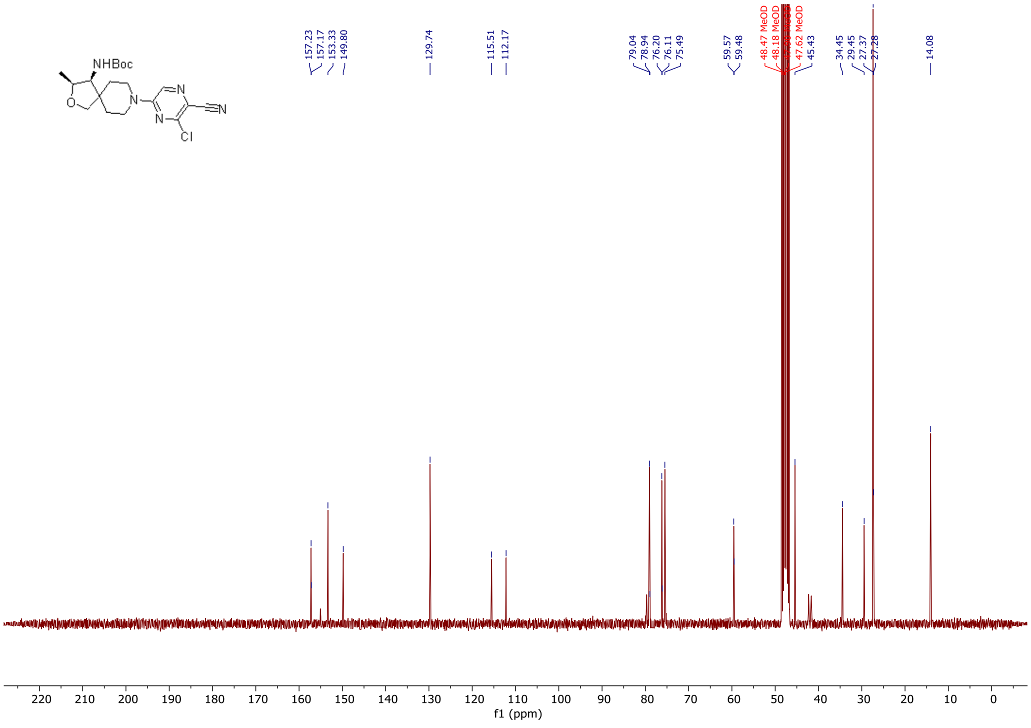

Compound **13** (^{1}H-NMR, MeOD, 300 MHz)

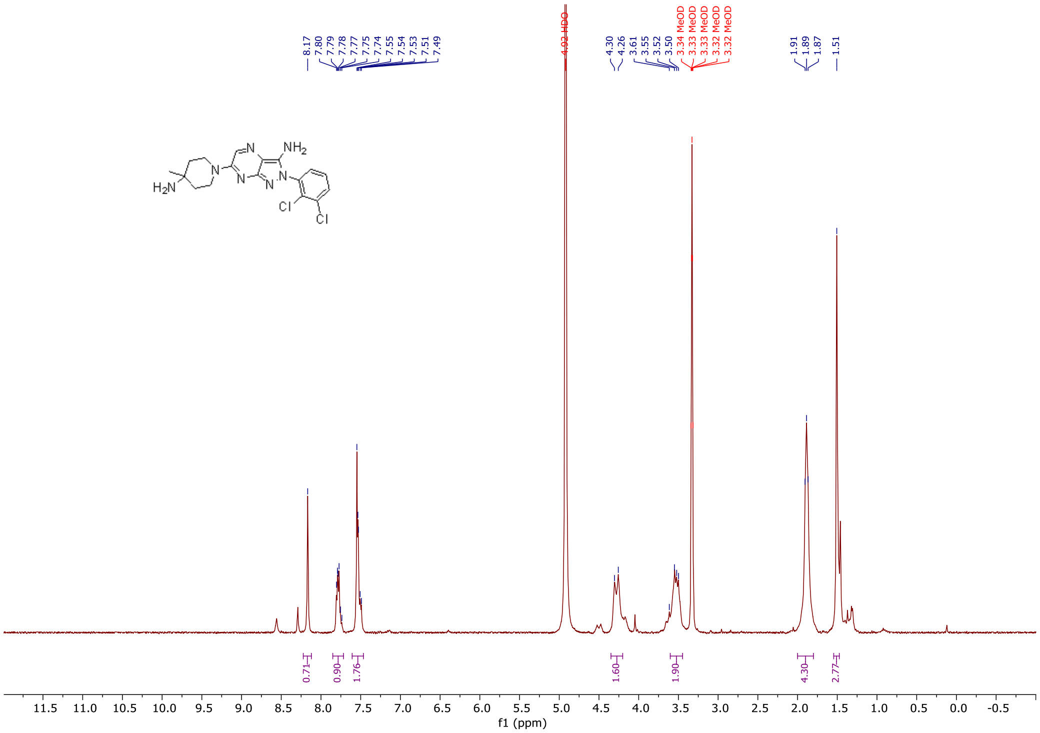

Compound **13** (^{13}C-NMR, MeOD, 75 MHz)

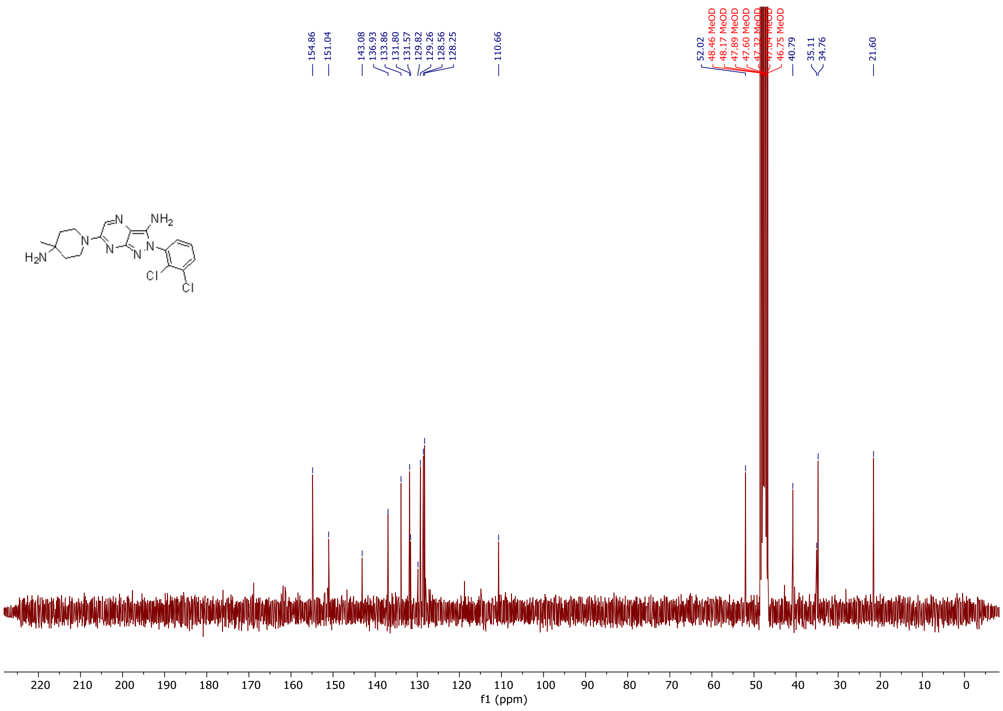

Compound **17e** (^{1}H-NMR, MeOD, 300 MHz)

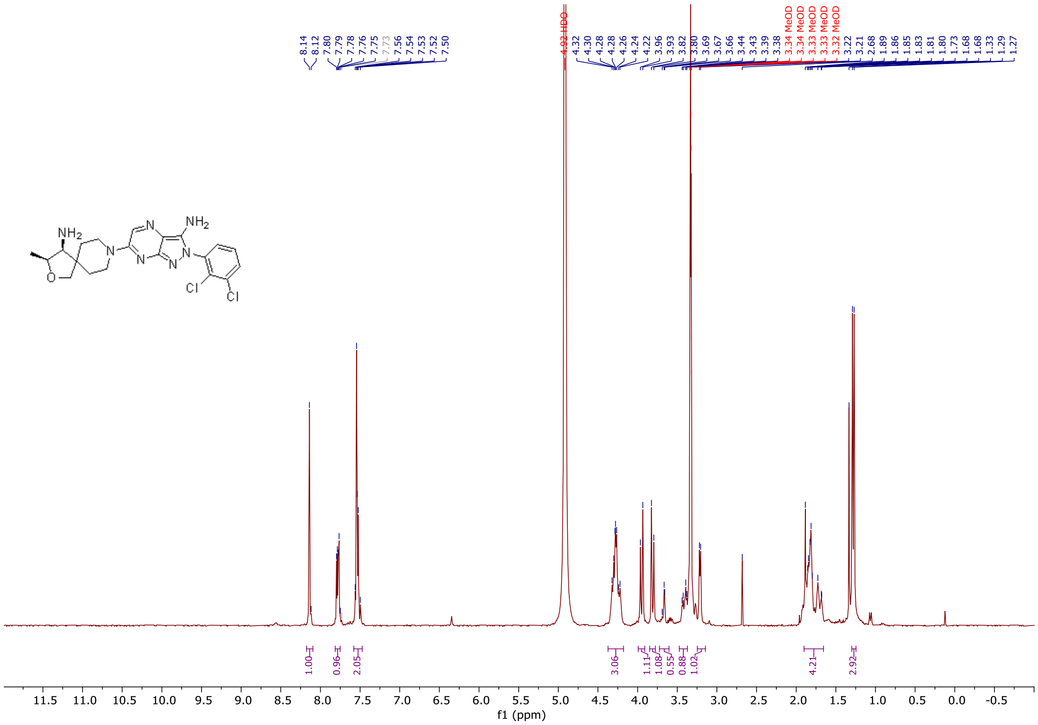

Compound **17e** (^{13}C-NMR, MeOD, 75 MHz)

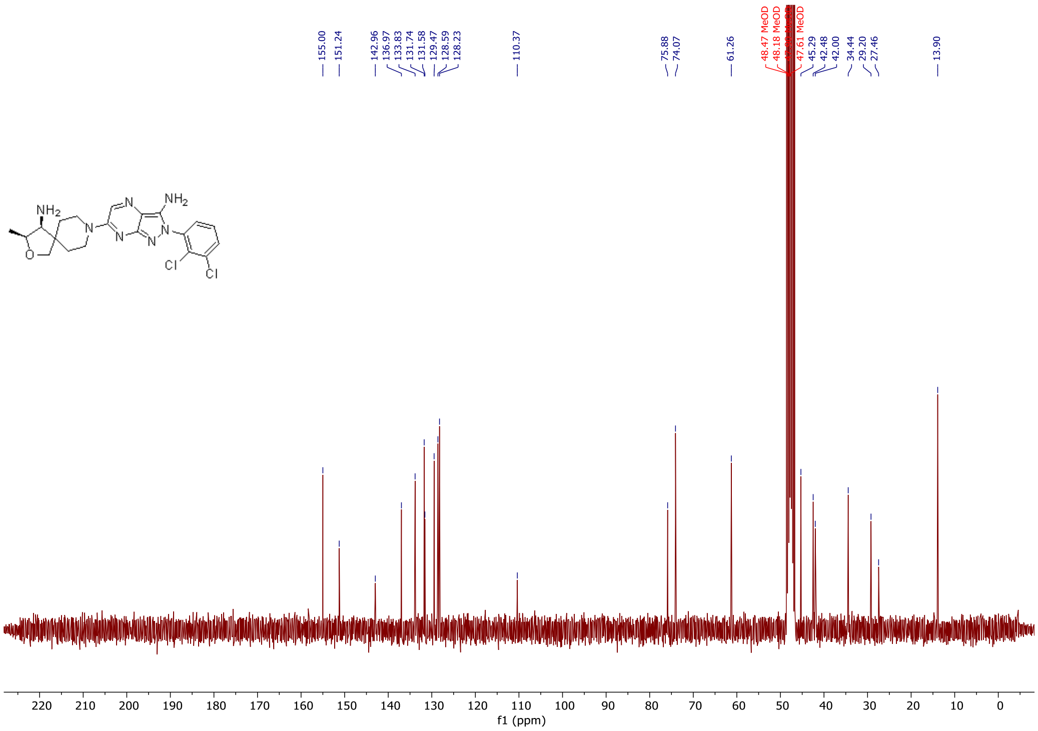

Compound **17f** (^{1}H-NMR, MeOD, 300 MHz)

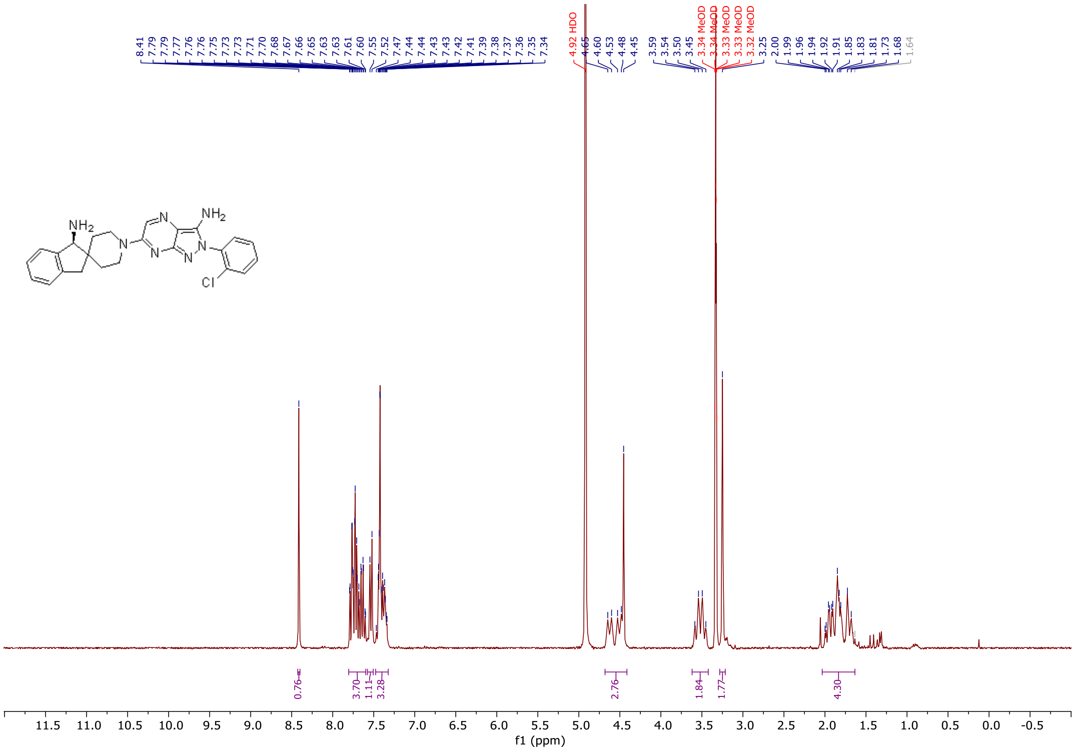

Compound **17f** (^{13}C-NMR, MeOD, 75 MHz)

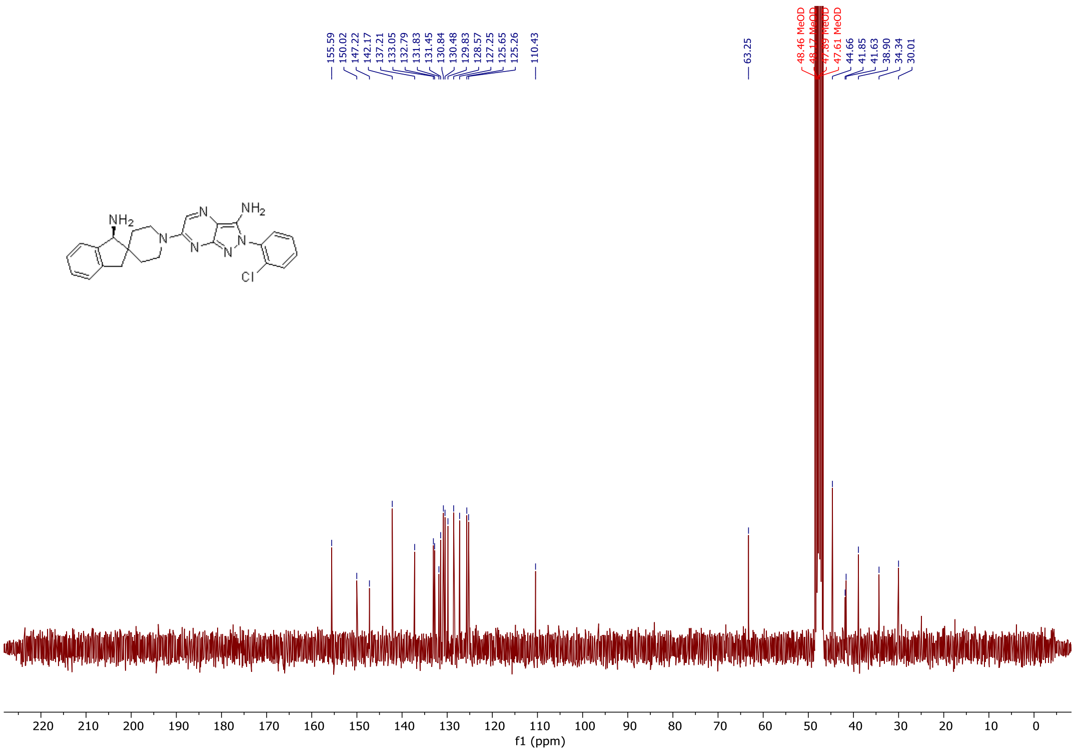

Compound **17g** (**^{1}H-NMR, MeOD, 600 MHz)**

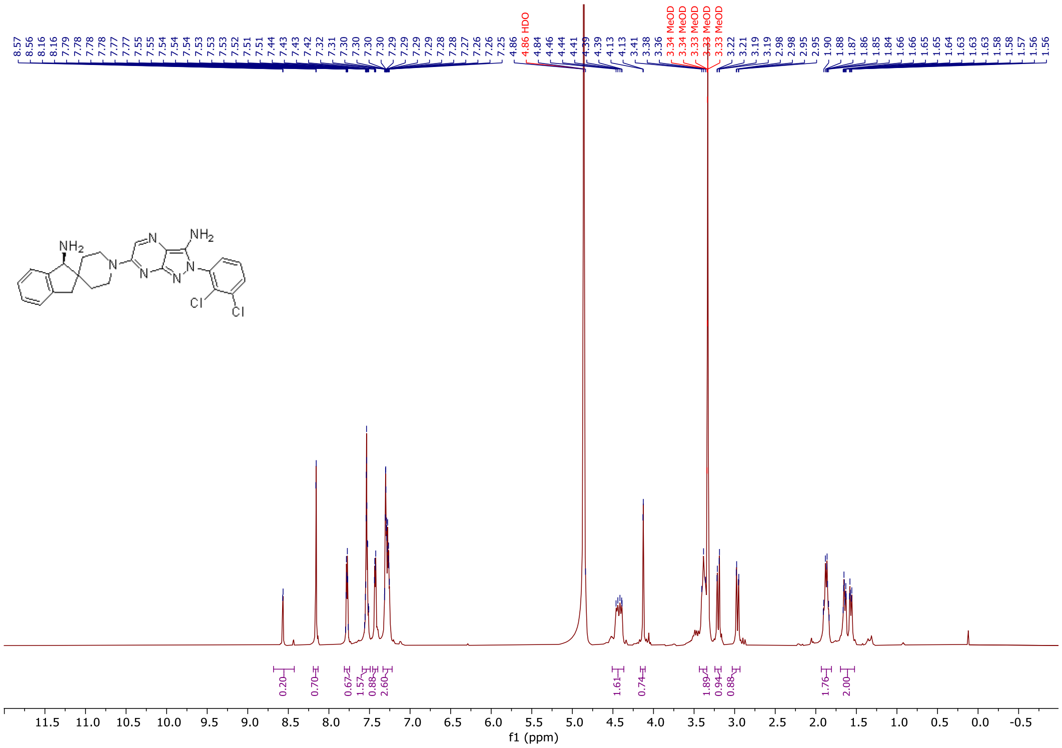

Compound **17g** (**^{13}C-NMR, MeOD, 151 MHz)**

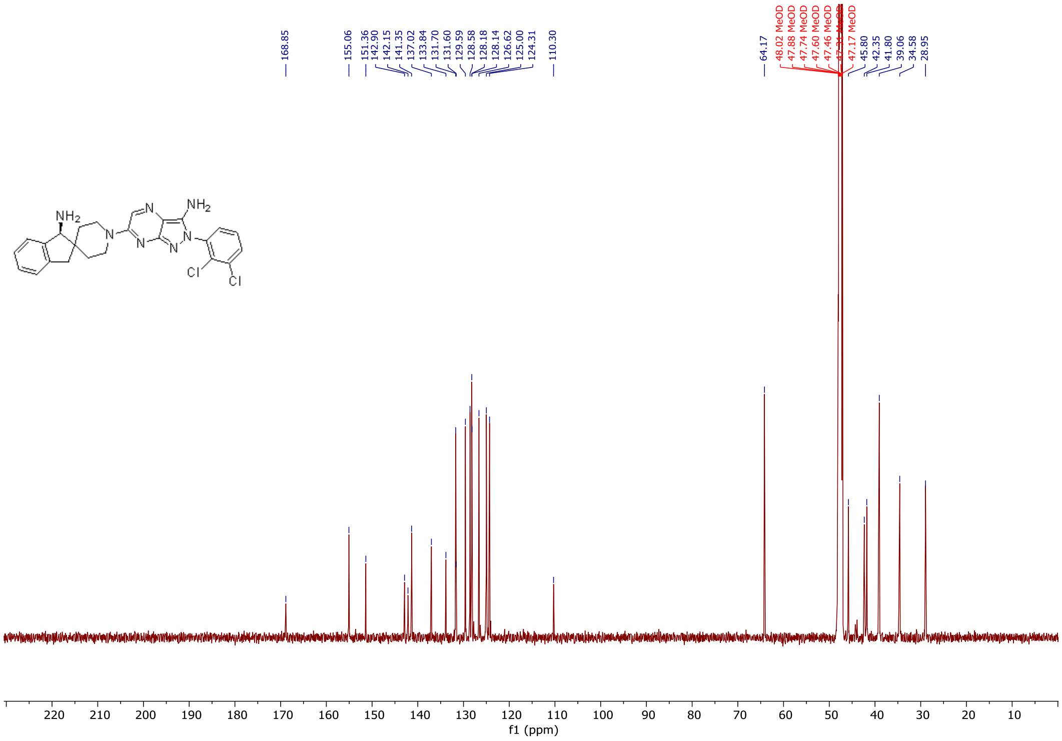

Compound **17h** (^{1}H-NMR, MeOD, 300 MHz)

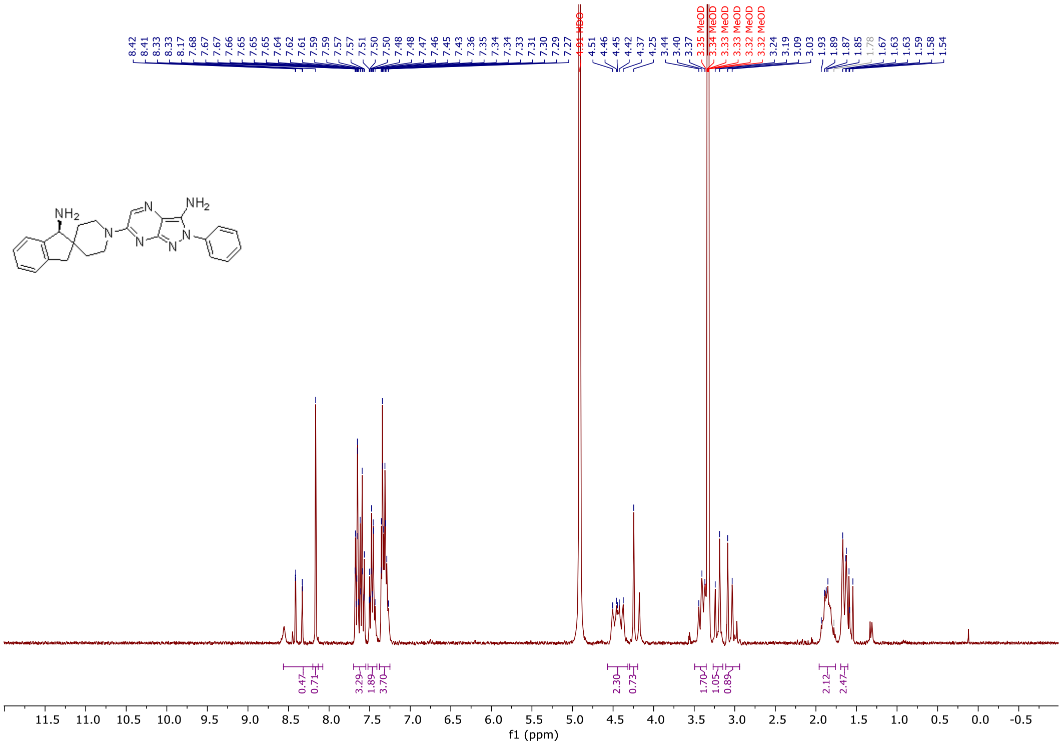

Compound **17h** (^{13}C-NMR, MeOD, 75 MHz)

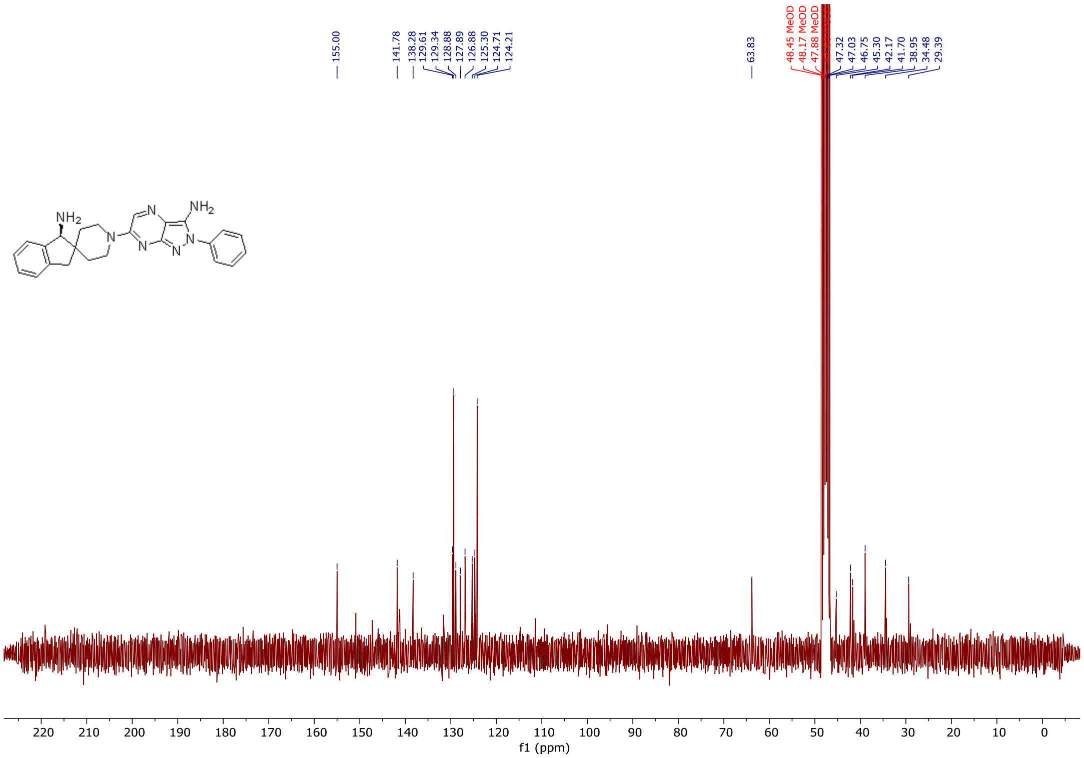

Compound **18** (^{1}H-NMR, MeOD, 600 MHz)

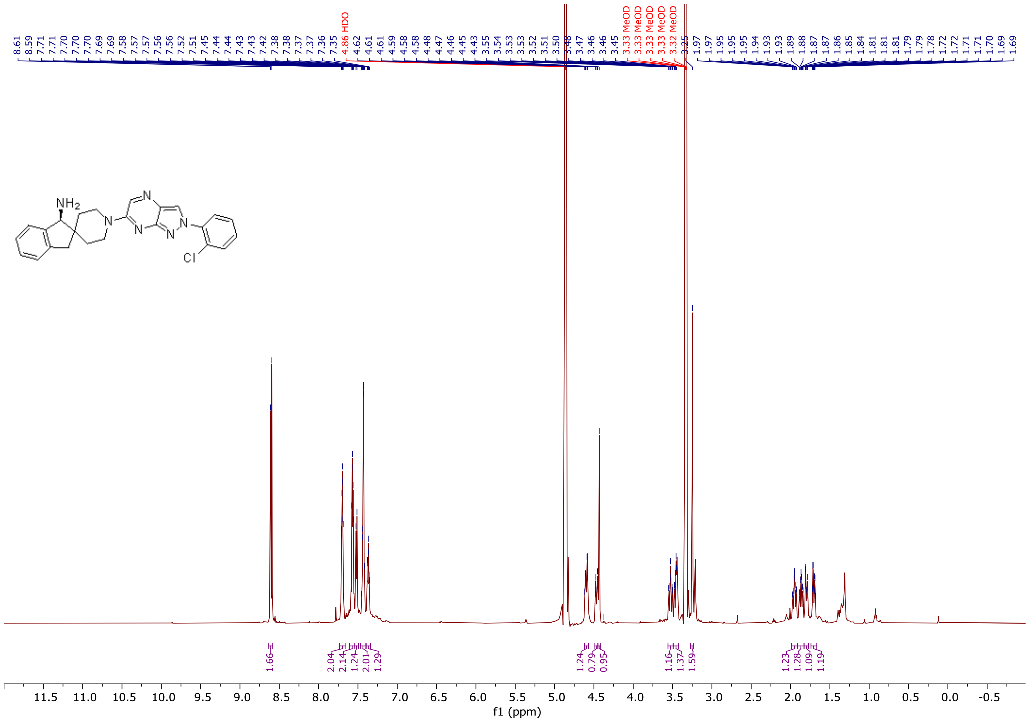

Compound **18** (^{13}C-NMR, MeOD, 151 MHz)

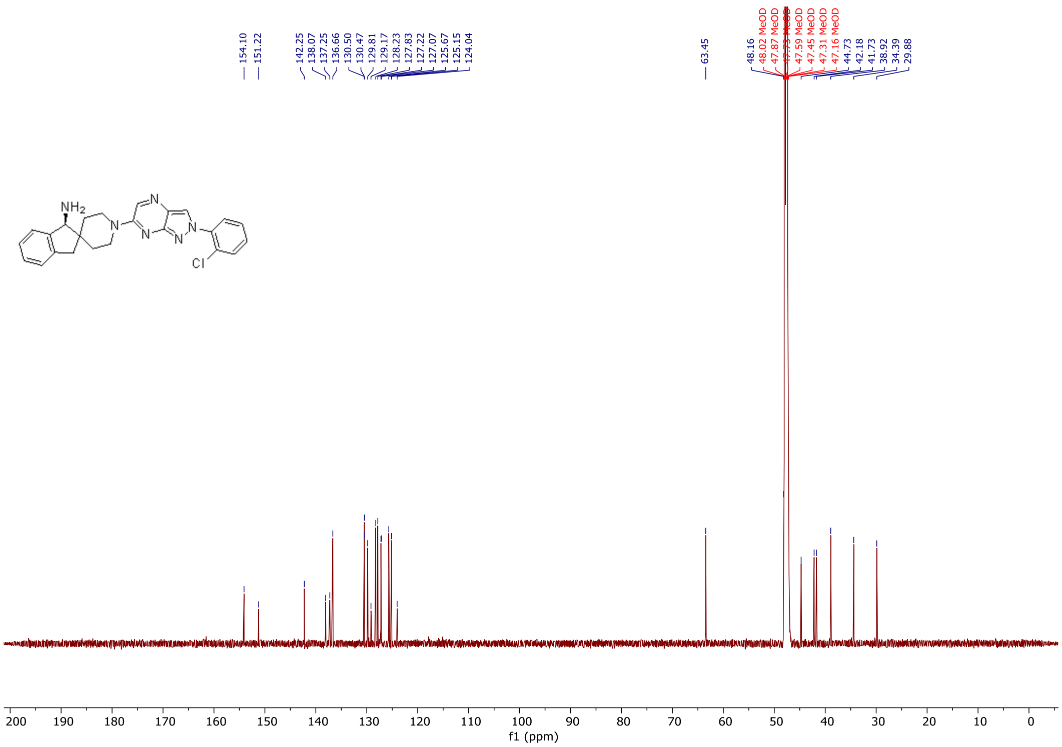

Supplementary Table S1: Data collection and refinement statistics

	$SHP2_{(2\text{-}525)}$ + compound 17g
**Data collection**	
Space group	$P2_12_12$
Cell dimensions	
a, *b*, *c* (Å)	56.5, 191.0, 52.2
α, β, γ (°)	90.0, 90.0, 90.0
Resolution (Å)*	48.6 - 1.87 (1.98-1.87)
R_{meas}* (%)	12.6 (171.6)
I / σ(I) *	8.2 (0.8)
Completeness (%)*	99.8 (99.8)
Redundancy*	3.7 (3.6)
CC(1/2) (%)*	99.7 (29.1)
No. reflections	330,585 (52,382)
No. unique reflections	89,938 (14,504)
**Refinement**	
R_{work}/ R_{free} (%)	0.184/0.229
No. atoms	
Protein	4068
Ligand/ion	58
Water	383
Mean B factor (Å^2)	45.9
R.m.s deviations	
Bond lengths (Å)	0.009
Bond angles (º)	0.970
Ramachandran outlier (%)	0.41
Mol/AU	1

* Data in the highest resolution shell are indicated in parentheses.

Superimposition of compound 21d onto compound 17g

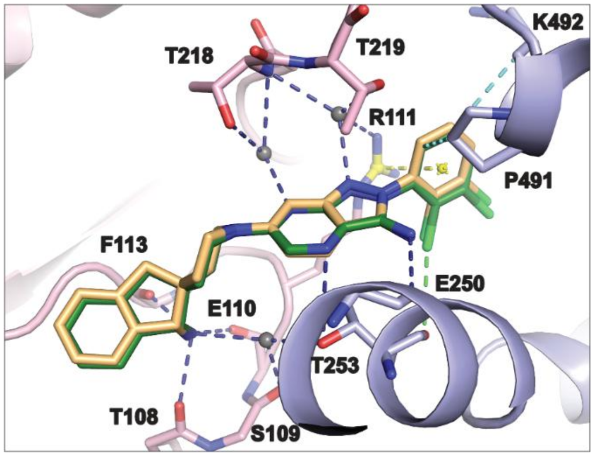

Supplementary Figure S3: Superimposition of compound **21d** (light orange) onto compound **17g** bound to SHP2 (green) demonstrates the loss of the critical contacts to Thr218 and Thr253 due to the lack of N-7 and N-4 in the pyrazine ring, respectively.

Reference

[1] R. Alsalim, P. Lindemann, M. P. Lopez-Alberca, S. Miksche, W. Czechtizky, N. Halland, M. Nazare, "A Palladium-Catalyzed Domino Reaction To Access 3-Amino-2H-indazoles from Hydrazines and 2-Halobenzonitriles", *Org Lett* **2020**, *22*, 7393-7396.

[2] M. Krug, M. S. Weiss, U. Heinemann, U. Mueller, "XDSAPP: a graphical user interface for the convenient processing of diffraction data using XDS", *Journal of Applied Crystallography* **2012**, *45*, 568-572.

[3] A. J. McCoy, R. W. Grosse-Kunstleve, P. D. Adams, M. D. Winn, L. C. Storoni, R. J. Read, "Phaser crystallographic software", *Journal of Applied Crystallography* **2007**, *40*, 658-674.

[4] P. Emsley, B. Lohkamp, W. G. Scott, K. Cowtan, "Features and development of Coot", *Acta Crystallographica Section D* **2010**, *66*, 486-501.

[5] P. D. Adams, P. V. Afonine, G. Bunkoczi, V. B. Chen, I. W. Davis, N. Echols, J. J. Headd, L.-W. Hung, G. J. Kapral, R. W. Grosse-Kunstleve, A. J. McCoy, N. W. Moriarty, R. Oeffner, R. J. Read, D. C. Richardson, J. S. Richardson, T. C. Terwilliger, P. H. Zwart, "PHENIX: a comprehensive Python-based system for macromolecular structure solution", *Acta Crystallographica Section D* **2010**, *66*, 213-221.
